# Systemic inflammation disrupts oligodendrocyte gap junctions and induces ER stress in a model of CNS manifestations of X-linked Charcot-Marie-Tooth disease

**DOI:** 10.1186/s40478-016-0369-5

**Published:** 2016-09-01

**Authors:** Margarita Olympiou, Irene Sargiannidou, Kyriaki Markoullis, Christos Karaiskos, Alexia Kagiava, Styliana Kyriakoudi, Charles K. Abrams, Kleopas A. Kleopa

**Affiliations:** 1 Neuroscience Laboratory, The Cyprus Institute of Neurology and Genetics and Cyprus School of Molecular Medicine, Nicosia, Cyprus; 2 Neurology Clinics, The Cyprus Institute of Neurology and Genetics and Cyprus School of Molecular Medicine, 6 International Airport Avenue, P.O. Box 23462, , 1683 Nicosia, Cyprus; 3 Department of Neurology and Rehabilitation, University of Illinois at Chicago, Chicago, USA

**Keywords:** CMT1X, Cx47, Cx43, LPS model, Oligodendrocytes, Gap junctions

## Abstract

X-linked Charcot-Marie-Tooth disease (CMT1X) is a common form of inherited neuropathy resulting from different mutations affecting the gap junction (GJ) protein connexin32 (Cx32). A subset of CMT1X patients may additionally present with acute fulminant CNS dysfunction, typically triggered by conditions of systemic inflammation and metabolic stress. To clarify the underlying mechanisms of CNS phenotypes in CMT1X we studied a mouse model of systemic inflammation induced by lipopolysaccharide (LPS) injection to compare wild type (WT), connexin32 (Cx32) knockout (KO), and KO T55I mice expressing the T55I Cx32 mutation associated with CNS phenotypes. Following a single intraperitoneal LPS or saline (controls) injection at the age of 40–60 days systemic inflammatory response was documented by elevated TNF-α and IL-6 levels in peripheral blood and mice were evaluated 1 week after injection. Behavioral analysis showed graded impairment of motor performance in LPS treated mice, worse in KO T55I than in Cx32 KO and in Cx32 KO worse than WT. Iba1 immunostaining revealed widespread inflammation in LPS treated mice with diffusely activated microglia throughout the CNS. Immunostaining for the remaining major oligodendrocyte connexin Cx47 and for its astrocytic partner Cx43 revealed widely reduced expression of Cx43 and loss of Cx47 GJs in oligodendrocytes. Real-time PCR and immunoblot analysis indicated primarily a down regulation of Cx43 expression with secondary loss of Cx47 membrane localization. Inflammatory changes and connexin alterations were most severe in the KO T55I group. To examine why the presence of the T55I mutant exacerbates pathology even more than in Cx32 KO mice, we analyzed the expression of ER-stress markers BiP, Fas and CHOP by immunostaining, immunoblot and Real-time PCR. All markers were increased in LPS treated KO T55I mice more than in other genotypes. In conclusion, LPS induced neuroinflammation causes disruption of the main astrocyte-oligodendrocyte GJs, which may contribute to the increased sensitivity of Cx32 KO mice to LPS and of patients with CMT1X to various stressors. Moreover the presence of an intracellularly retained, misfolded CMT1X mutant such as T55I induces ER stress under inflammatory conditions, further exacerbating oligodendrocyte dysfunction and pathological changes in the CNS.

**Electronic supplementary material:**

The online version of this article (doi:10.1186/s40478-016-0369-5) contains supplementary material, which is available to authorized users.

## Introduction

X-linked Charcot-Marie-Tooth (CMT) disease (CMT1X), associated with over 400 different mutations in the *GJB1* gene, is one of the commonest forms of inherited neuropathies (http://www.molgen.ua.ac.be/CMTMutations/) [8, 28]. *GJB1* encodes connexin32 (Cx32), a protein that forms gap junctions (GJs) both in Schwann cells in the peripheral nerves and in oligodendrocytes throughout the CNS, among other tissues [1, 59]. CMT1X patients present with slowly progressive weakness and atrophy usually starting in distal leg muscles, causing difficulty in running and frequently sprained ankles, typically beginning by 10 years of age. Males are earlier and more severely affected than heterozygous females who may be asymptomatic or may have a milder clinical form of the disease at an older age [26].

CMT1X mutations have been associated with clinical CNS phenotypes in addition to peripheral neuropathy, including chronic corticospinal tract dysfunction manifesting with spasticity, extensor plantar responses and hyperactive reflexes in patients with the A39V [38], T55I [50], M93V [7], R164Q [50], R164W [20], R183H [9], T191 frameshift [31] and L143P [30] mutations. Subclinical evidence of CNS involvement, such as abnormal brainstem auditory, visual, and motor evoked responses [6], is seen in a high proportion of cases [44, 45]. Acute transient encephalopathy, along with MRI changes, has been described in CMT1X patients carrying the M1L, T55I, R75W, E102del, R142W, R142Q, R164W, R164Q, C168Y and V177A mutations [49, 52, 65]. In most cases encephalopathy occurred under conditions of metabolic stress such as traveling to high attitudes [52], febrile illness [18, 58], hyperventilation [63] or concussion [2, 17]. CNS dysfunction caused by *GJB1* mutations is more common in children and young adults [2] without correlation to the stage and severity of the peripheral neuropathy.

The cellular mechanisms leading to CNS phenotypes in a subset of CMT1X patients remain unclear. Connexins are synthesized in the endoplasmic reticulum (ER), transported to the Golgi apparatus and then inserted as hexamers into the cell membrane [42]. Both in vivo and in vitro studies of CMT1X mutations have shown that many mutants are retained in the ER or Golgi with reduced or absent formation of GJ plaques at the cell membrane, [29, 75] and these missfolded mutants are effectively degraded by proteasomes and lysosomes [70]. Impaired formation of GJs by mutant Cx32 molecules and the loss of Cx32 function is responsible for the development of the neuropathy in most CMT1X mutations [26] with the exception of rare reports of toxic mutants associated with severe neuropathy phenotypes [33]. A gain-of-function mechanism for CNS manifestations has been considered both because patients with complete lack of the coding sequence have no subclinical CNS manifestations [16, 64] and because a disproportionate fraction of the mutations associated with the florid manifestations are represented in more than one pedigree, suggesting that specific mutations are particularly prone to cause this syndrome. Nevertheless, an ADEM-like CNS phenotype has been recently reported in a patient with start codon *GJB1* mutation [24] leading to complete loss of Cx32 synthesis [56], suggesting that loss of Cx32 function alone is sufficient to account for the CNS phenotypes in CMT1X.

Although trans-dominant effects of Cx32 mutants on co-expressed oligodendrocyte connexins Cx47 and Cx29 have been hypothesized, we found no interaction in vivo of two representative Cx32 mutants showing retention in the ER (T55I) or Golgi (R75W) with other connexins expressed in oligodendrocytes [57]. However, these mice were not subjected to stressful conditions as described in some CMT1X patients, and toxic effects only manifesting under stress conditions such as neuroinflammation may have been missed. Pro-inflammatory cytokines may disrupt glial GJs both by affecting connexins in astrocytes [10, 23], as well as in oligodendrocytes [36]. In the case of CMT1X patients and related models, this could further exacerbate the already tenuous GJ coupling of oligodendrocytes, leading to clinical manifestations.

To further clarify the cellular mechanisms underlying the CNS phenotypes in patients with CMT1X, we used a model of systemic inflammation induced by lipopolysaccharide (LPS) injection comparing wild-type (WT) mice, Cx32 knockout (KO) mice, and Cx32 KO expressing the T55I mutant of Cx32 (KO T55I). We hypothesized that dominant effects of the T55I mutant might only become manifest under stress conditions that mimic the metabolic and inflammatory events leading to encephalopathy in CMT1X patients. We found that a combination of cellular alterations, both mutant-related but also independent from the mutant, likely contribute to disrupt oligodendrocyte integrity.

## Materials and methods

### Experimental animals

Adult male (p40–p60) mice were used for this project and kept under standard housing conditions with a 12 hours (h) dark/light cycle and with food and water provided *ad libitum*. All experiments were performed in accordance with the animal care protocols approved by the Cyprus Government’s Chief Veterinary Officer according to EU guidelines. Three genotypic groups were used (*n* = 26 per group): Wild type (WT), connexin32 knockout (Cx32 KO), and Cx32 KO expressing the T55I mutant (KO T55I) associated with CMT1X. The KO T55I mutant was generated by pronuclear injection of the transgenic cassette described in [57] on a Bl6/N background and then crossed with the backcrossed Cx32 KO line. The WT was a 6 N and the Cx32 KO (originally generated by Prof. Klaus Willecke, University of Bonn, Bonn, Germany) was backcrossed at least 12 times to ensure a similar genetic background for the purpose of comparing the response to LPS. Only male animals were used for this study.

### Determination of Genotypes

Genotypes were identified by PCR of genomic tail DNA with specific primers for the Cx32 KO allele: Exon1F (5’- GACCACTCCCCCTACACAGA-3’), Cx32R (5’- CGCTGTTGCAGCCAGGCTGG-3’) resulting in a 732 bp PCR product (94 °C × 5 min, 40 cycles of 94 °C × 30 s, 57 °C × 30 s, 72 °C × 30 s and then 72 °C × 7 min) and Exon1F (5’-GACCACTCCCCCTACACAGA-3’), NeoR2 (5’-CTCGTCCTGCAGTTCATTCA-3’) resulting in a 721 bp PCR product (94 °C × 5 min, 35 cycles of 94 °C × 30 s, 56 °C × 30 s, 72 °C × 30 s and then 72 °C × 7 min). For the KO T55I, all primers specific for the Cx32 KO were used, and in addition the CNP1F (5’- TGTGGCTTTGCCCATACATA-3’), Cx32R (5’-CGCTGTTGCAGCCAGGCTGG-3’), resulting in a 732 bp PCR product (94 °C × 5 min, 40 cycles of 94 °C × 30 s, 57 °C × 30 s, 72 °C × 30 s and then 72 °C × 7 min) and Cx32F (5’- CGCTGTTGCAGCCAGGCTGG-3’), EGFPR1 (5’- GCTGAACTTGTGGCCGTTTA-3’), resulting in a 785 bp PCR product.

### LPS model

Lipopolysaccharide (LPS) from *Escherichia coli* (*E. coli*) (serotype 0127:B8) was purchased from Sigma Chemical (St. Louis, MO) and 150 μl (LPS stock concentration 1 μg/mL, 7.5 mg/Kg injected LPS) were injected once intraperitonealy (i.p) to induce a systemic inflammatory response. Sterile saline i.p injection was used as control treatment for all experiments. Animals were weighted daily until the day of sacrifice (7 days after injection) and were observed for any sickness behavior.

### ELISA

Approximately 100 μl of blood was collected from the vein with a glass micropipette, at different time-points (just before saline/LPS injection, as well as 4, 24 and 96 h after injection). For blood collection mice were positioned under warm light for five minutes prior to blood collection in order to increase blood flow and then placed in a restraining tube so that their head and body were restrained, leaving only the tail outside the tube. Using sharp scissors, approximately 1 cm of the tail was removed and blood was collected in a capillary tube as drops appeared. Pressure was applied to stop the bleeding and for further collection the original wound was re-opened by removing the clot. Following blood collection, blood was incubated at 37 °C for 20 min in the heat block, stored overnight at 4 °C, subjected to centrifugation at 3000 rpm for 10 min and then stored in aliquots at −80 °C. Expression levels of plasma pro-inflammatory cytokines, tumor necrosis factor alpha (TNF-α) and Interleukin-6 (IL-6) were evaluated in LPS and saline treated animals in order to confirm the induced inflammatory response upon LPS injection. ELISA was performed using the Mouse TNF alpha ELISA Ready-SET-Go!® (eBioscience) for the detection of mouse TNF-α and the Mouse IL-6 ELISA Ready-SET-Go!® (eBioscience) kit for the detection of mouse IL-6.

### Behavioral analysis

#### Foot slip test

The method of Britt et al. [11] modified for mice was used in order to assess motor co-ordination. Approximately 4 h after injection mice were placed in a 15 × 15 × 15 cm clear plexiglass box with a floor consisting of a metal wire grid with 1.25 cm spacing with a 1.25 cm grid suspended 1.25 cm above the floor. A trial consisted of 50 steps. If a misstep resulted in the hindlimb falling through the grid but the limb was withdrawn prior to touching the floor it was scored 1; if the limb touched the floor it was scored 2. A video camera was used to film the mice. Mice were acclimated by performing a trial on each of 3 consecutive days prior to injection and for 60 min in the box prior to testing.

#### Rotarod analysis

Four hours after saline/LPS injection mice were tested for motor balance and co-ordination. Animals were placed on a 3.5 cm diameter rod, rotating at a constant speed of 12 rpm and the time took for the animal to fall off was recorded using a timer. Mice were left to rest for 15 min and then tested at 20 rpm. Mice were trained on the rotarod for 3 trials of 3 consecutive days prior to injection. Mice were also acclimated to the testing room for 60 min before each session.

### Immunoblot analysis

For immunoblot analysis, saline- and LPS-injected animals (*n* = 4 per treatment condition from each genotypic group) were sacrificed 7 days after injection and fresh tissues (brainstem and cerebellum) were harvested and lysed in ice-cold RIPA buffer (10 mM sodium phosphate pH 7.0, 150 mM NaCl, 2 mM EDTA, 50 mM sodium fluoride, 1 % NP-40, 1 % sodium deoxycholate, and 0.1 % DSD) containing a mixture of protease inhibitors (Roche). Tissues were then sonicated 5 × 5 s and protein concentrations were measured with Nanodrop at λ = 280. Proteins (100 μg) from tissue lysates were fractionated by 12 % SDS-PAGE and then transferred to a Hybond-C extra membrane (GE Healthcare Bio-Sciences), using a semi-dry transfer unit (GE Healthcare Bio-Sciences). The membrane was blocked for 1 h at room temperature (RT) with 5 % non-fat skimmed milk in Tris-buffered saline containing 0.1 % Tween-20 (TBS-T). Immunoblots were then incubated with the following antibodies: rabbit anti-Iba1 (BioCare, diluted 1:500), rabbit anti-BiP (Santa Cruz, diluted 1:1000), mouse anti-Cx43 (Millipore, diluted 1:1000), rabbit anti-Cx47 (1:20,000, [47]) or mouse anti-MBP (Abcam, diluted 1:5000) in 5 % non-fat skimmed milk in TBS-T, overnight at 4 °C. Following three 15 min washes in TBS-T, immunoblots were incubated for 1 h at RT with an anti-mouse or anti-rabbit HRP-conjugated secondary antiserum (Jackson ImmunoResearch Laboratories) diluted 1:3000) in 5 % non-fat skimmed milk in TBS-T. All membranes were reprobed with either GAPDH (Santa Cruz Biotechnology, diluted 1:3000) or tubulin (Developmental Studies Hybridoma Bank diluted 1:4000) to demonstrate the loading. The bound antibody was visualized by enhanced chemiluminescence system (ECL, GE Healthcare Bio-Sciences) and band intensity was quantified using Tinascan software after normalizing each band to the GAPDH or tubulin band intensity.

### Immunohistochemistry

For immunofluorescence staining, saline- and LPS-injected animals (*n* = 5 per treatment condition from each genotypic group) were sacrificed 7 days after injection. Mice were anesthetized with Avertin according to institutionally approved protocols and then transcardially perfused with saline followed by addition of 4 % paraformaldehyde (PFA) in 0.1 M PB. Tissues (midbrain, brainstem, cerebellum and spinal cord) were harvested and fixed for 30 min followed by cryoprotection in 20 % sucrose in phosphate buffer (PB) overnight. Tissues were then embedded in optimum cutting temperature compound (OCT), placed in ice cold acetone and stored at −80 °C. Twelve μm thick sections were thaw-mounted onto glass slides, permeabilized in cold acetone (−20 °C for 10 min), and incubated for 1 h at RT with blocking solution (5 % bovine serum albumin - BSA) containing 0.5 % Triton-X. The primary antibodies diluted in blocking solution were incubated overnight at 4 °C: mouse Cx43 (Millipore, 1:200), CC1 (Calbiochem 1:50), GFAP (Sigma, 1:200), RT97 (Hybridoma Bank, 1:1000), MOG (Dr. Sara Piddlesden, Cardiff, 1:200) and MBP (Abcam, 1:500), and rabbit antisera against Cx47 (Invitrogen, 1:500), Iba1 (BioCare, 1:500), activated caspase-3 (BioVision, 1:100), Fas (Santa Cruz, 1:200), CHOP/GADD153 (Santa Cruz, 1:50), BiP (Santa Cruz, 1:50), Fibrinogen (Dako, 1:100) and Fibronectin (Dako, 1:500). Sections were then washed in PBS and incubated with rhodamine (TRITC) conjugated affinity purified goat anti-rabbit IgG and fluorescein (FITC) conjugated affinity purified goat anti-mouse IgG secondary antibodies (Jackson ImmunoResearch Laboratories, 1:500) for 1 h at RT. Cell nuclei were visualized with 4’, 6’-diamidino-2-phenylindole (DAPI) (Sigma-Aldrich). Slides were mounted with Dako fluorescent mounting medium and images were photographed under a Zeiss fluorescence microscope with a digital camera using the Zeiss Axiovision software (Carl Zeiss MicroImaging). For Cx47 localization some images were also taken with a Leica DMR confocal microscope (Leica Microsystems).

### Assessment of inflammatory cell infiltrates

Spinal cord roots and sciatic nerves of saline and LPS injected animals were also harvested as described above in order to assess the existence of inflammatory cell infiltrates (lymphocytes and macrophages) in the peripheral nervous system with immunohistochemistry. Rabbit antiserum against T-cell marker CD3^+^ (Santa Cruz, diluted 1:100) and fluorescein conjugated rat antiserum against macrophage marker CD68^+^ (Serotec, diluted 1:50) were used as primary antibodies.

### Morphometric analysis

Immunostained coronal sections of brainstem, cerebellum and spinal cord were visualized and captured with an AxioHR camera. Images of Iba1, MBP, MOG, and GFAP, immunostained sections were captured at 200× final magnification whereas images of connexin and CC-1 stained sections were captured at 400× final magnification. To assess microglial activation we measured the total area of Iba1 immunofluorescence and for myelin density evaluation we measured the total area of MBP immunofluorescence with ImageJ software, and results were presented as percentage of total image area. Oligodendrocyte numbers per total area were counted in CC-1 stained sections.

### Quantification of GJ plaques formed by Cx47 and Cx43

Quantification of GJ plaques (defined as a focal accumulation of connexin immunoreactivity and size set between 0.01 and 1 μm^2^ for Cx43, and between 0.01 and 2 μm^2^ for Cx47) was performed within a 9620 μm^2^ area using Image Pro 6.3 software in spinal cord gray and white matter and in brainstem sections stained with antibodies against Cx43 and Cx47 as described previously [36]. The total number of GJ plaques formed by Cx43 and Cx47 was measured. In addition, we counted the total number of oligodendrocytes labeled with the CC1 antibody in the same areas, and also quantified the number of Cx47 GJ plaques per oligodendrocyte in a fixed area of 30x30μm. All results of morphometric analysis were compared between LPS and saline treated mice in each genotype as well as between genotypes.

### RNA extraction and quantitative Real-Time PCR

RNA was isolated from the brainstem using the RNeasy Lipid Tissue Mini Kit (Qiagen, Germany) according to manufacturer instructions using the Qiazol Lysis Reagent followed by DNase treatment. RNA samples were quantified by spectrophotometry (Nanodrop ND_100) and subjected to reverse transcription (RT)-PCR (25 °C for 10 min, 48 °C for 30 min, and 95 °C for 5 min) using the TaqMan RT-PCR Reagents and a GeneAmp PCR System (Applied Biosystems, Singapore) (end volume of 40 μL). The expression levels of genes encoding Cx47, Cx43 and BiP were assessed by quantitative Real-Time PCR Analysis (hold at 55 °C for 2 min and at 95 °C for 10 min, followed by 40 cycles at 95 °C for 15 s and at 60 °C for 1 min) using a 7900HT Real-Time PCR System (Applied Biosystems) and Taq-Man^©^ Gene Expression Assays: Cx43: Mm01179639_s1; Cx47 Mm00519131_s1; BIP: Mm00517691_m1, Tubulin (Mm00726185_s1) was used as endogenous “house- keeping” control gene. Each sample was loaded in triplicate and contained 250 ng of cDNA, 1 μl of TaqMan Gene Expression Assay, and 10 μl of TaqMan Gene Expression Master Mix (end volume 20 μl). Expression levels in LPS and saline control mice were calculated after normalizing cycle thresholds against tubulin and presented as the fold induction value (2^–D DCt^) relative to naive control mice (mean ± standard deviation).

### Statistical analysis

All data were expressed as the mean, standard deviation SD; standard error of the mean (SEM), and statistical significance was assessed with the two-tailed Student’s *t*-test followed by Bonferroni's correction when multiple comparisons were performed, using Microsoft Excel software. A value of *p* < 0.05 was considered statistically significant.

## Results

### Establishment of the LPS-induced systemic inflammation model

The CNS phenotypes in CMT1X patients are frequently triggered by febrile illness [2, 58, 65]. In order to study this we developed a model of LPS-induced systemic inflammation. Intraperitoneal (i.p.) injection of LPS (150 μl) induced a systemic inflammatory response characterized by sickness behavior with transient mild weight loss (1.7 ± 0.64 gr at 24 h post injection), lethargy, isolation, and reduced mobility in the first 2 days after injection, followed by recovery of mobility back to normal levels. ELISA confirmed a marked increase of two pro-inflammatory cytokines, TNF-α and IL-6, 4 h after injection in peripheral blood of Cx32 KO LPS-injected as opposed to saline-injected mice (Additional file 1: Figure S1).

**Fig. 1:**
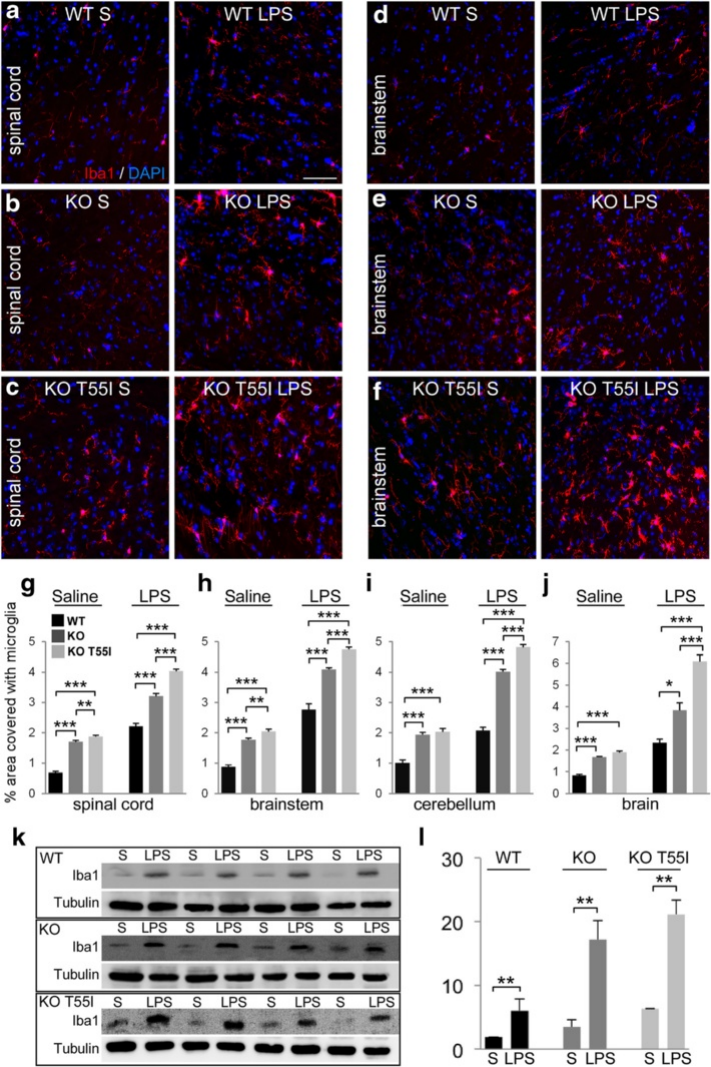
Diffuse CNS inflammation induced by systemic LPS injection. Images of fixed longitudinal sections of spinal cord (**a**–**c**) and brainstem (**d**–**f**) as indicated immunostained for microglial marker Iba1 (red) 1 week after injection. Nuclei are counterstained with DAPI (blue). LPS activates microglia in both CNS areas and in all genotypes (*WT = wild type; KO = Cx32 KO; KO T55I = Cx32 KO/T55I mutant*) compared to saline-injected mice. Scale bars in **a**–**f**: 50 μm. **g**–**j** Quantification of Iba1 immunofluorescence (% of total area) in different CNS areas (mean ± SEM) in *n* = 5 mice per group shows a significant increase in LPS-injected mice compared to controls in all areas studied across all genotypes. KO T55I tissues show the highest levels of activated microglia (data shown in Additional file 3: Table S1). Immunoblot analysis of Iba1 levels (band at 17 kDa) in brainstem lysates from WT, KO and KO T55I-(LPS) and saline-injected (S) mice, as indicated (**k**) and quantification (**l**) of Iba1 levels (specific band intensity, normalized for loading with tubulin re-blotting) confirms a significant elevation in LPS compared to saline injected mice of all genotypes, with the highest elevation in KO T55I mice (only significant values are shown, Student’s *t*-test, *:*p* < 0.05, **:*p* < 0.01, ***:*p* < 0.001, Bonferroni corrected)

Systemic inflammation was associated with a diffuse CNS inflammatory response, demonstrated by immunohistochemistry for Iba1, a microglia marker. Compared to saline injected control mice, in LPS-injected mice microglia were diffusely activated in all CNS areas examined including the spinal cord, brain, brainstem and cerebellum (Fig. 1a–f and Additional file 2: Figure S2). Quantification of Iba1 immunoreactivity (*n* = 5 animals per treatment group) confirmed that microglia were significantly activated in the CNS of LPS treated mice compared to controls, in all genotypes and in all areas studied (Fig. 1g–j). Even at baseline in saline groups there was increased Iba1 immunoreactivity in T55I KO compared to KO and WT mice suggesting that more activated microglia are present (Additional file 3: Table S1). The immunostaining findings were further corroborated by immunoblot analysis of Iba1 levels. Compared to saline treated mice, LPS-injected mice showed significantly elevated Iba1 levels in all genotypes studied, including the WT, Cx32 KO, and KO T55I mice (Fig. 1k, l). The highest LPS-induced Iba1 increase was found in KO T55I mice, both by immunostaining and by immunoblot suggesting that inflammation in exacerbated by the presence of the T55I mutant, which is retained in the ER [29, 57], as confirmed by double labeling with ER marker BiP (Additional file 4: Figure S3). In contrast to CNS, we could not detect any significant inflammatory changes in the peripheral nervous system after LPS injection in these 2-month old Cx32 mutant mice, in which peripheral nerve pathology begins after the age of 3 months [5, 57, 60]. Immunostaining for macrophages (CD68) and T-cells (CD3) failed to show any differences compared to saline controls in sciatic nerves or lumbar spinal roots (Additional file 5: Figure S4). Thus, Cx32 KO mice show a higher degree of baseline CNS inflammation and a stronger CNS inflammatory reaction to LPS-induced inflammation compared to WT mice, while the presence of the ER-retained T55I mutant on Cx32 KO background further exacerbates these abnormalities.

### LPS-induced neuroinflammation impairs motor performance and coordination

**Fig. 2:**
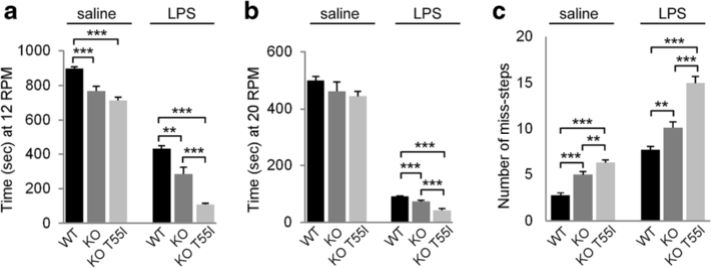
Impaired motor performance in LPS-injected mice. Bar charts representing the effect of LPS-induced neuroinflammation on motor performance examined by rotarod test at 12 rotations per minute (RPM) (**a**) and at 20 RPM (**b**) as well as by Foot-slip test (**c**) in WT, Cx32 KO, and KO T55I mice, as indicated. Time required for the animal to fall off the rotarod was recorded using a timer. Saline injected animals of all three genotypes were able to stay much longer on the rotarod compared to LPS injected animals at both speeds tested (**a**, **b**). Even at baseline levels KO T55I performed worse that simple KO animals, while WT animals outer-performed KO animals both in control and in LPS groups (data shown in Additional file 6: Table S2). Foot-slip analysis (**c**) revealed that LPS-injected animals showed more missteps compared to saline-injected controls of all three genotypes. Additionally, more missteps were shown by Cx32 KO compared to WT mice, and by T55I KO compared to simple KO mice, both at baseline and after LPS (Student’s *t*-test, *:*p* < 0.05, **:*p* < 0.01, ***:*p* < 0.001, Bonferroni corrected)

We investigated motor performance and coordination comparing LPS and saline treated animals by using two behavioral tests, the foot slip and rotarod test (*n* = 18 per genotype, *n* = 9 saline, *n* = 9 LPS). When tested 4 h after injection LPS-treated animals from all three genotypes fell off the rotarod much sooner than the saline treated animals at both speeds tested (Fig. 2a–b). They also took longer to complete the 50 steps trial during the foot-slip test with a higher number of miss-steps compared to animals receiving saline in all three genotypes (Fig. 2c). Among LPS treated mice, KO T55I mice showed the worst performance compared to Cx32 KO and WT mice, and in turn Cx32 KO mice performed worse than WT mice. Thus, LPS-induced inflammation affected significantly the motor performance in all genotypes but more severely the Cx32 KO expressing the T55I mutant than the simple KO or the WT groups (Additional file 6: Table S2). Interestingly, even at baseline in saline treated groups, Cx32 KO and even more T55I KO mice showed worse motor performance than WT animals. Thus, Cx32 KO and even more T55I KO mice show deficits in motor performance and coordination compared to WT mice already at baseline, but in addition a more severe impairment of their performance after LPS-induced inflammation, indicating a higher vulnerability under stress conditions.

### LPS induced neuroinflammation does not cause demyelination or blood-brain barrier disruption in Cx32 mutant mice

To clarify whether the observed inflammatory changes and impaired motor function could be caused by demyelination in the CNS of Cx32 mutant mice, we examined myelination by immunohistochemistry and immunoblot analysis. Immunostaining for myelin basic protein (MBP) and the axonal marker RT97, as well as for myelin oligodendrocyte glycoprotein (MOG), showed preservation of myelin immunoreactivity in different areas of the CNS, including the brain at the level of the corpus callosum (Additional file 7: Figure S5a–l), cerebellum and spinal cord (data not shown) in LPS-injected mice without significant change compared to saline-injected controls. In addition, immunoblot analysis confirmed that MBP levels assessed in brainstem lysates were not significantly altered in LPS-injected compared to control mice from all three genotypes (Additional file 7: Figure S5m–o). Thus, demyelination is unlikely to contribute to the observed phenotype of LPS-injected Cx32 mutant mice.

Given the increased CNS inflammation in Cx32 KO and KO T55I mice we also examined whether blood-brain barrier (BBB) disruption could play a role in CNS phenotypes in Cx32 mutant mice following systemic inflammation induced by LPS. We therefore examined expression of fibrinogen and fibronectin, two major BBB markers [53], on fixed brain tissues comparing LPS to saline treated tissues for each genotype. We found no evidence of BBB disruption in KO or KO T55I animals injected with LPS compared to WT and saline controls (Additional file 8: Figure S6).

### LPS-induced systemic inflammation disrupts oligodendrocyte gap junction formation in the CNS

**Fig. 3:**
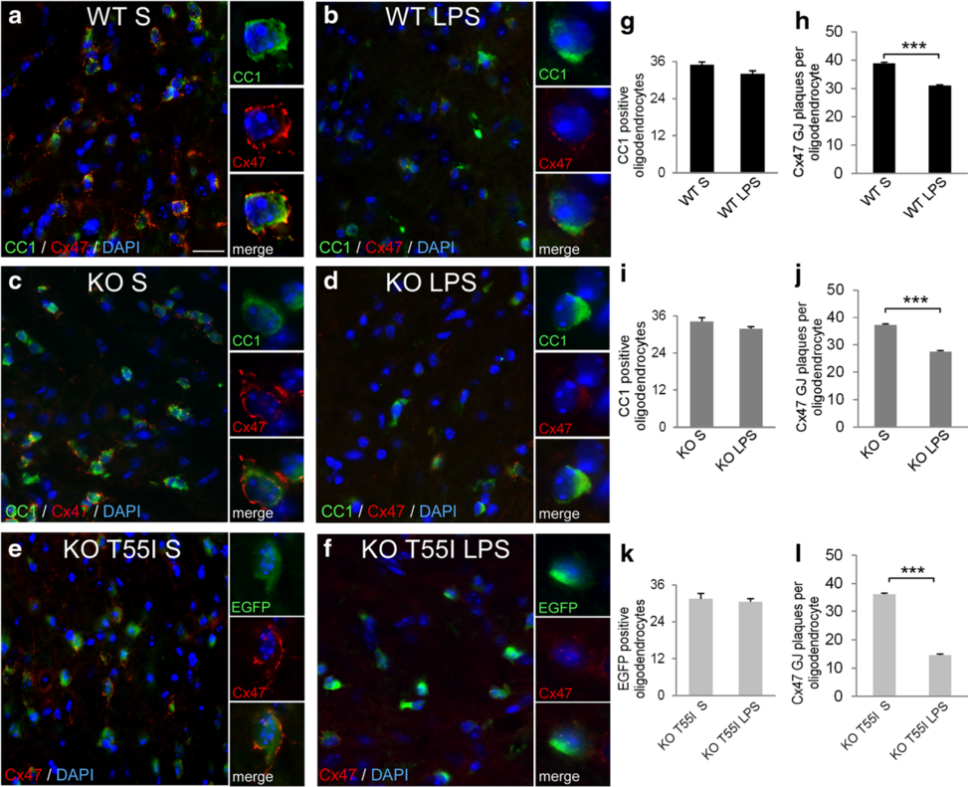
Disruption of oligodendrocyte gap junctions (GJs) in inflamed brainstem. **a**–**f** Coronal brainstem sections immunostained for oligodendrocyte marker CC1 (green) and Cx47 (red) with nuclear DAPI staining (blue). In KO T55I tissues all oligodendrocytes transgenically express enhanced green fluorescent protein (EGFP) and were not stained with CC1. There is a reduction of Cx47 formed GJ plaques at oligodendrocyte cell bodies and proximal processes in LPS-injected mouse tissues (**b**, **d**, **f**) compared to saline controls (**a**, **c**, **e**) associated with increased intracellular signal of Cx47 suggesting diffusion (insets in **d**, **f**). Scale bars in **a**–**f**: 10 μm. Counts of GJ plaques per oligodendrocyte confirm a significant reduction in LPS tissues compared to controls in all genotypes (**h**, **j**, **l**), whereas total oligodendrocyte numbers remain unchanged in LPS compared to control groups (**g**, **i**, **k**) (Student’s *t*-test, *:*p* < 0.05, **:*p* < 0.01, ***:*p* < 0.001)

In order to evaluate whether LPS induced neuroinflammation disrupts oligodendrocytic GJ formation, which in Cx32 KO mice depends mainly on Cx47, we investigated by double immunofluorescence staining the expression of Cx47 in CC1-stained oligodendrocytes in different CNS areas including the gray and white matter of the spinal cord (Additional file 9: Figure S7 and Additional file 10: Figure S8), brainstem (Fig. 3), and cerebellum (data not shown). Cx47, which is normally expressed in cell bodies and proximal processes of all oligodendrocytes throughout the CNS [27], was markedly reduced in inflamed brainstem, cerebellum and spinal cord, compared to saline controls in all three genotypes studied. This disruption of Cx47-formed GJs was associated with diffuse Cx47 immunoreactivity intracellularly in oligodendrocytes (Fig. 3, Additional file 9: Figure S7, Additional file 10: Figure S8 and Additional file 11: Figure S9). Loss of Cx47 GJ plaques was not due to loss of oligodendrocytes since double staining with the oligodendrocyte marker CC1 along with quantification of Cx47 GJ plaques *per oligodendrocyte* confirmed that oligodendrocytes were not reduced in numbers, but showed severely reduced Cx47 GJ plaques per individual cell, in all CNS areas from LPS-injected mice examined.

Although we did not observe any significant reduction in the numbers of CC1-positive oligodendrocytes in LPS tissues we further investigated by double immunostaining with CC1 and caspase-3 whether LPS induced any oligodendrocyte apoptosis in WT or Cx32 mutant mice. This analysis showed that caspase-3 immunoreactivity was not increased in the CNS of LPS-injected WT, Cx32 KO, or T55I KO mice compared to saline controls (Additional file 12: Figure S10). Thus, LPS-induced inflammation causes loss of Cx47 GJ plaques in oligodendrocytes but no oligodendrocyte loss or apoptosis, up to 1 week after injection.

### LPS-induced neuroinflammation disrupts astrocyte to oligodendrocyte gap junctions

**Fig. 4:**
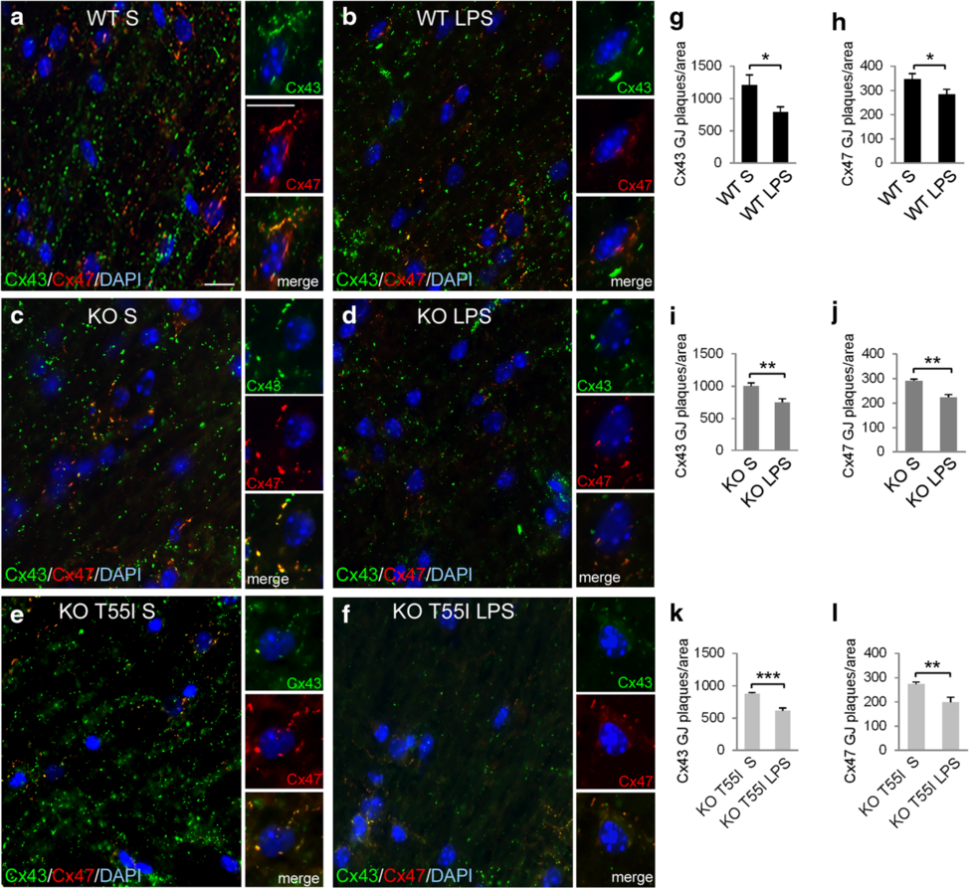
Disruption of astrocyte to oligodendrocyte GJs in inflamed spinal cord white matter (WM). **a**–**f** Fixed longitudinal spinal cord WM sections immunostained for astrocytic Cx43 (green) and Cx47 (red) with nuclear DAPI staining (blue) show reduced overall Cx43 immunoreactivity in LPS-injected spinal cord tissues (**b**, **d**, **f**) of all genotypes compared to saline controls (**a**, **c**, **e**). Fewer GJ plaques are formed by both Cx43 as well as Cx47 at oligodendrocyte cell bodies and proximal processes, which are often colocalized in control more than in LPS treated mice. In oligodendrocytes from LPS treated mice there is often a diffused signal of Cx47 intracellularly (inset in **f**). Scale bars in **a**–**f**: 10 μm. Counts of GJ plaques formed by Cx43 (**g**, **I**, **k**) as well as by Cx47 (**h**, **j**, **l**) confirm a significant reduction in LPS treated mice of all genotypes (Student’s *t*-test, *:*p* < 0.05, **:*p* < 0.01, ***:*p* < 0.001)

To further clarify the cause of the extensive loss of Cx47 GJs observed in LPS treated mice, we also examined the expression and GJ formation by Cx43, the main astrocytic partner of Cx47. Double immunostaining for Cx43 and Cx47 revealed a marked loss of Cx43 formed GJs in both gray and white matter of the spinal cord in LPS-injected mice compared to controls. There was decreased immunoreactivity of Cx43 along with a patchy appearance in all areas examined, most severely in KO T55I LPS spinal cord (Fig. 4 and Additional file 13: Figure S11, Additional file 14: Figure S12 and Additional file 15: Figure S13). Cx43 GJ plaques that normally appear denser around oligodendrocyte cell bodies and colocalize with Cx47 were markedly reduced, associated with reduction of Cx47 GJ plaques and diffuse Cx47 cytoplasmic signal. This disruption of Cx43 expression was not associated with either astrocyte loss or astrogliosis, as shown by double immunostaining with the astrocyte marker GFAP, which demonstrated preserved pattern of astrocyte immunoreactivity (Additional file 16: Figure S14). To further corroborate these findings, we counted the total number of Cx43 as well as Cx47 GJ plaques in spinal cord white (Fig. 4) and gray matter (Additional file 13: Figure S11), as well as in the brainstem (Additional file 14: Figure S12). This analysis confirmed the significant reduction of Cx43 GJ plaque numbers similar to Cx47 in all examined CNS areas from LPS-injected mice compared to controls from all genotypes.

**Fig. 5:**
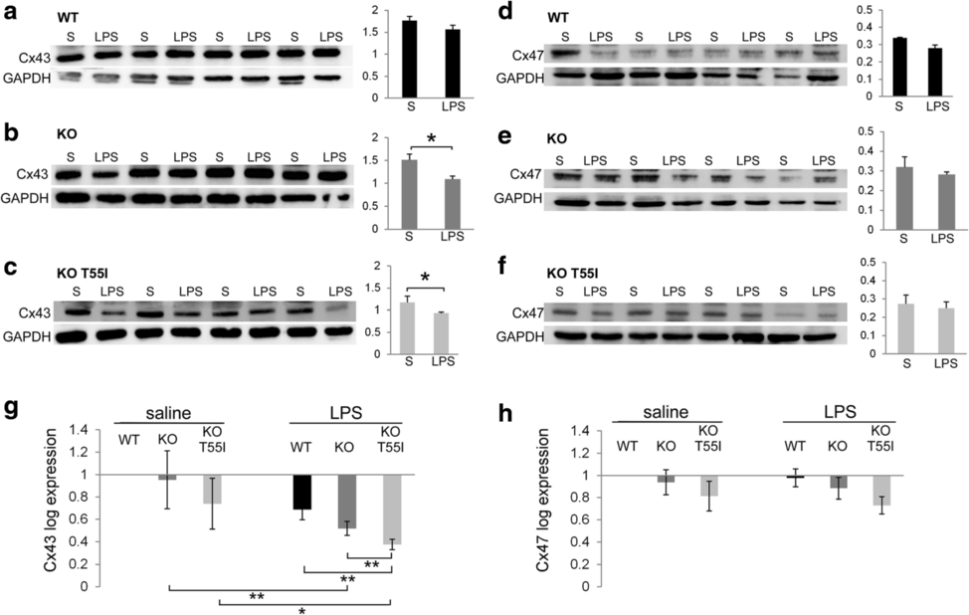
Loss of Cx43 in the brainstem following LPS-injection. Immunoblot analysis (blots and quantification diagrams) of Cx43 (**a**–**c**) and Cx47 (**d**–**f**) levels in brainstem lysates from groups (*n* = 4 per genotype and treatment group) of WT (**a**, **d**), Cx32 KO (**b**, **e**) and KO T55I (**c**, **f**) LPS or saline (S) injected mice, as indicated. All blots were re-probed for GAPDH for loading control and normalized bands were measured using Tinascan software. Compared to saline controls, Cx43 levels (specific band at 43 kDa) are reduced in the brainstem of LPS-injected Cx32 KO and KO T55I mice. In contrast, Cx47 levels do not show any significant changes in LPS treated mice in any of the genotypes (**d**, **e**, **f**) (*:*p* < 0.05, Student’s *t*-test). Real-time PCR analysis of Cx43 (**g**) and Cx47 (**h**) expression in LPS- compared to saline-treated mice shows a significant reduction of Cx43 expression in LPS injected tissues of KO and KO T55I groups, while the reduction in the WT group was non-significant. The KO T55I group showed the strongest reduction after LPS compared to the other genotypes. In contrast, Cx47 mRNA levels showed no significant changes in LPS-treated mice (Student’s-test, *:*p* < 0.05, **:*p* < 0.01, ***:*p* < 0.001, Bonferroni corrected)

Quantitative immunoblot analysis of Cx43 levels in brainstem lysates showed that Cx43 was significantly reduced in Cx32 KO and KO T55I LPS groups compared to saline controls (Fig. 5a–c), whereas in LPS treated WT mice the Cx43 reduction was not significant. Thus, there is a significant disruption or increased recycling/ degradation of Cx43 expression and GJ formation in astrocytes that may play a role in the loss of Cx47 GJs in oligodendrocyte of Cx32 KO mice in the setting of LPS-induced neuroinflammation. In contrast, Cx47 protein levels assessed by immunoblot analysis were not significantly altered in LPS-treated mice (Fig. 5d–f), suggesting that Cx47 was initially retracted from GJs but remained in the cytoplasm. Indeed, as shown below the loss of oligodendrocytic Cx47 GJ plaques in this LPS model of CMT1X appears to follow the loss of Cx43 GJs in astrocytes (at least at the level of transcription).

### Downregulation of Cx43 precedes the loss of Cx47 in LPS-injected Cx32 mutant mice

To clarify which of the connexin partners at A/O GJs is affected first by LPS-induced neuroinflammation in Cx32 mutant mice, we examined their expression by Real-time PCR analysis. These experiments revealed that Cx43 mRNA levels were significantly reduced in CNS tissues from KO and KO T55I LPS-injected mice compared to controls, while a non-significant reduction was also observed in the WT group. Comparison of baseline Cx43 mRNA levels between the three genotypes at baseline (saline) showed no significant difference. However, among LPS-treated groups Cx43 expression was significantly more reduced in KO T55I than in simple KO and the WT groups (Fig. 5g). We also analyzed Cx47 mRNA expression levels comparing LPS-injected to control mice in all genotypic groups both at baseline and after LPS injection. Although we observed a small reduction in the expression of Cx47 in the T55I KO groups, there was no significant difference compared to WT or KO mice or in LPS compared to control groups (Fig. 5h). Thus, astrocytic Cx43 appears to be downregulated under inflammatory conditions with reduction of GJ channels. Loss of Cx43 is associated with reduction of oligodendrocyte Cx47 GJs and A/O connectivity, and diffusion of Cx47 away from the cell membrane, since Cx47 mRNA and protein levels are not reduced. These alterations appear to be more severe in the Cx32 KO T55I mutant than in the simple Cx32 KO mouse.

### The expression of the ER-retained T55I mutant triggers ER-stress under inflammatory conditions

**Fig. 6:**
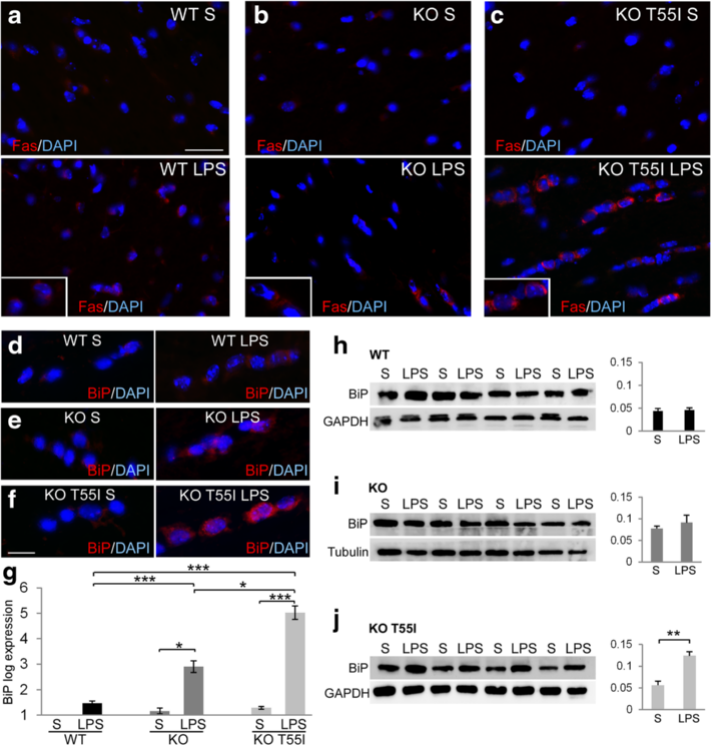
Systemic inflammation induces ER-stress most severely in the CNS of Cx32 KO T55I mutant mice. Images of brainstem sections focusing on white matter areas from saline (S) or LPS treated WT (**a**), Cx32 KO (**b**) and KO T55I (**c**) mice as indicated, immunostained with the ER-stress and pro-apoptotic marker Fas (red). Cell nuclei are stained with DAPI (blue). Higher magnification details of oligodendrocytes are shown in insets. Fas immunoreactivity is increased in LPS tissues, most prominently in KO T55I brainstem (**c**). Likewise, spinal cord longitudinal sections immunostained for the ER-stress marker BiP (red) (**d**–**f**) show increased immunoreactivity in LPS-treated mice compared to saline controls, especially in KO T55I mice. Scale bars in a-c: 30 μm; in **d**–**f**: 10 μm. **g** Real-time PCR analysis of BiP expression comparing LPS to saline groups from WT, KO and KO T55I animals, as indicated, shows that BiP mRNA is elevated in LPS tissues compared to saline controls in Cx32 KO and even more in KO T55I groups but not in WT mice. Direct comparison of LPS groups from all three genotypes confirms elevation of BiP expression in Cx32 KO and KO T55I mice compared to WT mice, and in KO T55I significantly more than in the simple KO group. **h**–**j** Immunoblot analysis of BiP levels (BiP specific band at 74 kDa) in brainstem tissue lysates (*n* = 4 mice per genotype and treatment group) from LPS compared to saline treated WT (**h**), KO (**i**) and KO T55 (**j**) mice as indicated, shows significant elevation of BiP levels only in the KO T55I LPS group. Blots were re-probed for tubulin or GAPDH for loading control and normalized BiP band intensity was quantified with Tinascan software (Student’s-test, *:*p* < 0.05, **:*p* < 0.01, ***:*p* < 0.001, Bonferroni corrected)

To further examine whether the presence of the T55I mutant, which is known to be retained intracellularly in the ER in vitro and in vivo [29, 57], could cause any additional cellular stress exacerbating the observed LPS-induced changes, we analyzed the expression of early ER-stress markers CHOP, Fas and BiP by immunohistochemistry, and further examined BiP expression by immunoblot and Real-time PCR. Immunostaining for the apoptotic marker Fas, which is induced by early ER stress events [67], revealed increased CHOP and Fas immunoreactivity mostly in white matter oligodendrocytes of LPS treated KO T55I mice compared to their saline controls and to a lesser degree in KO and WT mice (Fig. 6a–c, Additional file 17: Figure S15). Likewise, BiP immunoreactivity was increased in spinal cord white matter in LPS treated mice but most prominently in KO T55I (Fig. 6d–f). Real-time PCR analysis confirmed at the mRNA level that BiP expression was significantly increased in the inflamed CNS of LPS-treated Cx32 KO mice, but this increase was much more pronounced in KO T55I mice (Fig. 6g). Quantitative immunoblot results revealed that BiP protein levels were significantly elevated in the brainstem of KO T55I LPS-treated compared to saline control mice but not in WT or Cx32 KO mice (Fig. 6h–j). Thus, neuroinflammation appears to cause ER stress in oligodendrocytes, and this is exacerbated in Cx32 KO mice compared to WT mice, while the presence of the ER-retained T55I mutant increases even further the ER stress response.

## Discussion

Our study provides further insights into the cellular and molecular mechanisms underlying the intriguing CNS phenotypes in patients with CMT1X. First, we show that in the absence of Cx32 in Cx32 KO mice there is a higher vulnerability to inflammation, since the associated downregulation of astrocytic Cx43 leads to reduction of Cx47-formed O/A GJ channels and impaired oligodendrocyte connectivity to the glial GJ network. This mechanism is independent from the presence Cx32 mutants. Second, we demonstrate that the presence of the ER-retained T55I mutant in a Cx32 KO background further increases oligodendrocyte vulnerability to inflammatory stress, through an increased ER-stress response. This mechanism could be additive to the disruption of A/O GJs, leading to even more severe CNS dysfunction.

The most common trigger of the CNS phenotype reported in CMT1X patients besides high altitude travel was a febrile systemic illness such as upper respiratory infection, fever of uncertain cause, pneumonia or gastroenteritis in at least half of all cases (reviewed by [2]). In order to reproduce the CNS phenotype induced by systemic inflammation in CMT1X patients, we generated a model of systemic inflammation induced in Cx32 mutant mice by intraperitoneal injections of LPS. We confirmed the generalized inflammatory response by detecting transient elevation in blood of two pro-inflammatory cytokines, TNF-α and IL-6, as shown in other studies [14]. LPS activates liver cells to produce cytokines in the periphery, which in turn activate humoral and neural communication pathways [12, 55, 62] and induce glial cells in the CNS to produce the same inflammatory cytokines. Activation of microglia, the resident immune cells of the brain, results in synthesis of additional TNF-α and other cytokines and leads to persistent neuroinflammation [54]. Likewise, in our study we observed diffusely activated microglia in different CNS areas including the cerebrum, cerebellum, brainstem and spinal cord and Iba1 immunoblots from brainstem lysates confirmed increased microglial activation induced by systemic LPS injection. Thus, LPS treatment provides a relevant model for the CNS phenotypes in CMT1X patients.

The LPS model described here was characterized by early sickness behavior and transient weight loss, similar to the phenotype reported in other models [19]. In addition, we examined motor behavior by rotarod and foot-slip test behavioral analysis, showing a marked impairment in LPS-treated mice compared to saline controls as shown in similar studies [62]. Moreover, the comparison of the three genotypes showed that Cx32 KO mice are more vulnerable to neuroinflammation than WT mice, and KO T55I show even more vulnerability than do simple KO mice. Thus, LPS-induced neuroinflammation affects motor performance even more in the presence of the T551 mutant, suggesting additional gain of function mechanisms that could explain the CNS phenotypes in some CMT1X patients.

Despite the diffuse response of the innate immunity in the CNS, we found no disruption of BBB, inflammatory cell infiltrates in the peripheral nervous system, oligodendrocyte loss, or demyelination in Cx32 mutant mice, indicating that these are unlikely mechanisms in CNS phenotypes occurring in CMT1X patients. Our results are therefore in agreement not only with the clinical course in CMT1X patients, who show early recovery, but also with the available MRI studies that have shown transient, nearly symmetric, diffuse T2 and fluid-attenuated inversion recovery (FLAIR) hyperintensities and restricted diffusion signal abnormalities, but no contrast enhancement and no typical demyelinating lesions [2, 49, 65]. The clinical and MRI characteristics of CNS involvement in CMT1X patients are in fact consistent with transient disruption of the gap junctional communication between oligodendrocytes and astrocytes leading to inability of these cells to regulate fluid exchange.

Astrocytes appear to play a role in the link between neuroinflammation or metabolic stress and CNS phenotypes in CMT1X as they are closely connected to oligodendrocytes through GJs. Oligodendrocytes express Cx47 throughout the CNS, and in many areas also Cx32 [27]. Besides their participation in A/O GJs, Cx32 and Cx47 also form homotypic channels between oligodendrocytes. Cx29 is a third oligodendrocyte connexin that appears to form only hemichannels and does not participate in intercellular GJs [3]. Astrocytes express Cx43 in both gray and white matter and Cx30 mainly in the gray matter, connecting with other astrocytes through homotypic (Cx43/Cx43 and Cx30/Cx30) GJs as well as with oligodendrocytes with heterotypic (Cx43/Cx47 and Cx30/Cx32) GJs [4, 22, 43, 48, 73]. Among these channels, Cx43/Cx47 GJs play the major role especially in the white matter.

Several experimental mouse models have been generated to clarify the role of each of these astrocyte and oligodendrocyte connexins in the CNS. Single KOs showed minimal CNS pathology leading to the overall conclusion that there is partially overlapping function of connexins in both cell types. However, when more than one connexin and type of GJ channel is disrupted, severe demyelinating pathology develops. Thus, mice lacking either Cx32 or Cx47 develop minimal CNS pathology and no obvious CNS phenotype, but loss of both oligodendrocyte connexins in Cx32/Cx47 double KO mice leads to severe and early CNS demyelination [41, 46]. Likewise, deletion of both astrocyte connexins (Cx30 and Cx43) [35], or at least one partner of each of the two A/O GJ types, either Cx43 and Cx32 [40], or Cx30 and Cx47 [69] results in severe demyelination and vacuolation because in each case all A/O GJs are completely disrupted. In contrast to these double KO models with congenital and complete lack of oligodendrocyte GJ channels, in our LPS model induced in Cx32 deficient mice there was only partial reduction of Cx47 GJs after completed development, which did not cause demyelination. This underscores the important role of Cx47 in oligodendrocytes with expression as starting at earlier stages of their development and differentiation [41, 46], as early as P7 [66], including in oligodendrocyte precursor cells [37].

Of particular relevance to the current study and the mechanism of CNS phenotypes in CMT1X is the Cx32/Cx43 double KO model, which revealed that in the absence of Cx32 GJs (as in CMT1X patients) oligodendrocytes depend exclusively on Cx43/Cx47 GJs. When Cx43 is also disrupted, the stability of Cx47 on oligodendrocyte cell membrane is impaired leading to loss of Cx47 formed GJs, and consequently to complete disconnection of oligodendrocytes [40]. In our LPS model we observe a clear downregulation of Cx43 in astrocytes with early reduction of mRNA levels. In contrast, there are no significant changes in Cx47 mRNA and protein levels, despite reduction of Cx47 GJ plaques and diffusion in the cytoplasm. This pattern of secondary disruption of Cx47 GJ plaques following the downregulation of astrocytic Cx43 during acute inflammation has also been shown in previous studies using the EAE model [36]. As in the EAE model, loss of Cx47 GJs was not associated with loss of oligodendrocytes, further supporting the secondary mechanism of Cx47 diffusion into the cytoplasm [40]. The downregulation of Cx43 in the LPS model likely reflects a non-specific astrocytic reaction to diverse CNS injury, including inflammation, and has been shown in EAE [10, 36, 61], ischemia [32] and abscess [23] models and is likely mediated by pro-inflammatory cytokines [13, 15, 21]. Thus, it is plausible to hypothesize that in CMT1X patients, in whom oligodendrocyte GJ connectivity depends only on Cx43/Cx47 GJs, downregulation of Cx43 as part of an astrocyte reaction to inflammatory or metabolic stress will disrupt Cx47 in oligodendrocytes and lead to transient encephalopathy. Our previous studies in EAE models showed that Cx43 downregulation is a transient reaction followed by re-expression at later stages [36], and this would be in keeping with the reversibility of CNS phenotypes in CMT1X patients. Nevertheless, inflammation could also directly affect Cx47 expression independently of astrocyte reaction and Cx43 loss, since pro-inflammatory cytokines have been shown to induce ER-stress response in oligodendrocytes [34].

Cx32 KO mice with or without the presence of the T55I mutant showed a worse phenotype than WT mice. One explanation is the higher CNS inflammatory load reflected in the amount of microglia activation. This would also explain why Cx43 was more severely decreased in these mutants, although not an oligodendrocyte connexin. A pro-inflammatory environment associated with elevated cytokine levels at baseline has been recently documented in Cx32/Cx47 dKO mice suggesting that connexin deficiency in oligodendrocytes drives CNS inflammation independently of external immune triggers [74]. The exacerbated phenotype of Cx32 mutant mice following LPS treatment may also result from effects of inflammation on neurons and axons directly or indirectly, for example through astrocyte dysregulation of synaptic function [51], independently of the effects in oligodendrocytes. Previous studies showed exacerbated axonal loss in Cx32 KO compared to WT mice after EAE induction [36], which may result from disturbed signaling [68] and energy supply [72], as well as axonal neurofilament dephosphorylation [71] in the absence of Cx32 GJs along myelinated fibers. Thus, axons of Cx32 KO mice may have increased vulnerability to inflammatory stress.

The next question is why the presence of the T55I mutant exacerbates all CNS manifestations in our LPS model, including the inflammatory, behavioral, and connexin abnormalities. Although our previous studies in Cx32 KO T55I mice failed to show a dominant effect beyond the simple KO phenotype under normal conditions [57], LPS-induced neuroinflammation affected more severely the KO T55I than the “simple” KO and WT mice. Thus, the presence of the T55I mutant may render oligodendrocytes more vulnerable to inflammation. We believe that this is because the T55I mutation causes retention of misfolded Cx32 in the ER leading to ER-stress response especially under inflammatory conditions, which in turn exacerbates further oligodendrocyte homeostasis. LPS induced a significant elevation of BiP expression especially in KO T55I mice, as opposed to WT and Cx32 KO mice, although Cx32 KO mice also showed a significantly higher ER-stress response compared to WT mice. Previous studies have shown that upon TNF-α release, microglia activation and ER stress are induced in the CNS and that ER stress blockers suppress the induced inflammation [39]. Involvement of ER stress has also been demonstrated in LPS-induced inflammation and inhibition of ER stress ameliorated LPS-induced lung injury through modulation of several cytokine pathways [25]. In addition we found increased Fas immunoreactivity induced by LPS injection mostly in KO T55I LPS treated mice compared to simple KO or WT. ER stress induces Fas and mitochondrial apoptosis pathways through elevation of cytosolic calcium [67]. In our study, both the ER stress marker BiP and CHOP, along with the pro-apoptotic marker Fas, were mostly elevated in the presence of the T55I mutant suggesting that under stress conditions intracellularly retained Cx32 mutants may exacerbate ER stress upon neuroinflammation, leading to the development of CNS phenotypes in CMT1X patients. This effect may be additive to the disruption of A/O GJs described above, making CNS manifestations more likely in patients with intracellularly retained mutants as initially hypothesized [29], although they can also occur in the absence of any Cx32 [56].

## Conclusion

In conclusion, the work presented here provides evidence that LPS-induced systemic and CNS inflammation causes oligodendrocyte dysfunction associated with disruption of the main A/O GJs resulting from downregulation of astrocytic Cx43 and diffusion of Cx47. Moreover, there is an exaggerated ER stress response related to the intracellularly retained Cx32 mutant. Our study provides a relevant model and a mechanistic explanation for the development of CNS phenotypes in CMT1X. In patients with Cx32 mutations the already compromised oligodendrocyte homeostasis causing subclinical manifestations at baseline conditions, may decompensate under conditions of metabolic and inflammatory stress producing the transient encephalopathy manifestations.

## Additional files

Additional file 1: Figure S1. 1 LPS induces IL-6 and TNF-α increase in Cx32 KO mice. a-b: Graphs representing concentration course of IL-6 before (0 h) as well as 4, 24, and 96 h after saline (a) or LPS (b) injection as measured at 450 nm with ELISA. Saline did not cause any significant systemic inflammation (baseline inflammation: 0.14 pg/mL, 4 h after injection: 0.23 pg/mL), whereas a marked increase 4 h after LPS injection (935.1 pg/mL) was found. c-d: Levels of TNF-α before (0 h) as well as after (4, 24, 96 h) saline (c) and LPS (d) injection; saline did not cause any inflammatory response (baseline: 0.21 pg/mL, at 4 h: 0.22 pg/mL) whereas LPS injection caused a marked TNF-α increase (4 h after injection: 22.08 pg/mL). (TIF 12301 kb) Additional file 2: Figure S2. LPS causes diffuse CNS inflammation in the cerebellum and cerebral cortex. Images of fixed coronal sections of cerebellum (a-f) and cerebral cortex (g-l), as indicated, immunostained for microglial marker Iba1 (red). Cell nuclei are counterstained with DAPI (blue). Microglia are diffusely activated in both CNS areas and in all genotypes in LPS (b, d, f, h, j, l) compared to saline (a, c, e, g, i, k) injected mice. Scale bars: 50 μm. (TIF 7715 kb) Additional file 3: Table S1. Quantification of Iba1 immunoreactivity in different CNS areas of saline and LPS treated WT, Cx32 KO (KO) and T55I KO mice. (DOCX 22 kb) Additional file 4: Figure S3. Localization of the T55I Cx32 mutant in the endoplasmic reticulum in oligodendrocytes. This is an image of spinal cord white matter longitudinal sections from a Cx32 KO T55I mutant mouse (saline control) double labeled with antibodies to Cx32 (green) and ER marker BiP (red). Cell nuclei are stained with DAPI (blue). Overview image is shown on the left and higher magnification images of cells in separate channels are shown on the right. The T55I mutant is retained intracellularly in oligodendrocytes and colocalizes in the perinuclear cytoplasm with the ER marker, indicating retention in the ER. No GJ-like plaques are formed by the mutant on the cell membrane. Scale bar: 10 μm. (TIF 16462 kb) Additional file 5: Figure S4. LPS injection does not cause inflammatory changes in the peripheral nervous system. Fixed longitudinal sections of sciatic nerves (a-f) and lumbar spinal roots (g-l) from LPS-injected and saline control mice from all three genotypes as indicated, immunostained with T-cell marker CD3 (red) and macrophage marker CD68 (green). Cell nuclei are stained with DAPI (blue). There is no difference in inflammatory cell immunoreactivity between treatment groups in any of the genotypes. No CD3+ cells are seen, whereas 1–2 CD68+ macrophages are present in all tissues regardless of the genotype and treatment condition. Scale bars in a-f: 30 μm; in g-l: 20 μm. (TIF 50275 kb) Additional file 6: Table S2. Results of behavioral testing in saline control and LPS treated WT, Cx32 KO (KO) and T55I KO mice. (DOCX 22 kb) Additional file 7: Figure S5. Lack of CNS demyelination in LPS-injected Cx32 mutant mice. a-l: Images of brain sections at the level of the corpus callosum immunostained with axonal marker RT97 (green) in combination with myelin marker MBP (red) (a-f) or with myelin marker MOG (g-l) and DAPI nuclear staining (blue). There is no apparent alteration of myelin immunoreactivity in the brains of LPS-injected mice (b, d, f, h, I, l) compared to saline controls (a, c, e, g, j, k) from all three genotypes, as indicated. Scale bar: 50 μm. m-o: Immunoblot analysis of MBP levels in brainstem tissue lysates show no significant change induced by LPS injection (LPS) compared to saline-injected mice (S) in WT (m), Cx32 KO (n) or KO T55I (o) groups as indicated. All blots were re-probed for tubulin to demonstrate the loading, and quantification of normalized MBP band intensity is shown next to each blot. (TIF 19222 kb) Additional file 8: Figure S6. LPS induced neuroinflammation does not cause blood brain barrier (BBB) disruption in Cx32 mutant mice. These are images of fixed coronal brain sections, immunostained with blood brain barrier markers fibronectin (green) (a-f) or fibrinogen (red) (g-l) and counter stained with DAPI (blue). There is no apparent disturbance of BBB integrity in the brain of LPS treated (b, d, f, h, j, l) compared to saline treated (a, c, e, g, i, k) mice from any of the three genotypes, as indicated. Linear fibrinogen immunoreactivity in brain parenchyma represents small blood vessels. Scale bar: 50 μm. (TIF 9451 kb) Additional file 9: Figure S7. LPS disrupts oligodendrocyte GJs formed by Cx47 in spinal cord gray matter. a-f: Fixed longitudinal spinal cord gray matter sections immunostained with oligodendrocyte marker CC1 (green) and Cx47 (red) and counterstained with DAPI (blue). In KO T55I tissues all oligodendrocytes transgenically express EGFP and were not stained with CC1. Cx47 immunoreactivity and GJ plaque formation at oligodendrocyte cell bodies and proximal processes is reduced in LPS-treated mice (b, d, f) of all genotypes compared to their saline controls (a, c, e). Scale bar: 20 μm. Counts of Cx47 GJ plaques per individual oligodendrocyte shows a significant reduction in LPS compared to saline treated mice (h, j, l), whereas the number of CC1/EGFP-positive oligodendrocytes per genotype as indicated shows no significant reduction in LPS compared to saline treated mice (Student’s *t*-test, *:*p* < 0.05, **:*p* < 0.01, ***:*p* < 0.001). (TIF 8996 kb) Additional file 10: Figure S8. LPS disrupts oligodendrocyte Cx47 GJs in spinal cord WM. a-f: Fixed longitudinal spinal cord WM sections immunostained for CC1 (green) and Cx47 (red) and counterstained with DAPI (blue). In KO T55I tissues all oligodendrocytes transgenically express EGFP and were not stained with CC1. Cx47 immunoreactivity and GJ plaque formation at oligodendrocyte cell bodies and proximal processes is reduced in LPS treated mice of all genotypes (b, d, f) compared to their saline controls (a, c, e), with increased cytoplasmic immunoreactivity insets in d, f). Scale bar: 10 μm. Quantification of CC1/EGFP-positive oligodendrocytes per genotype as indicated (g, i, k) shows no significant reduction in LPS treated compared to saline groups, whereas quantification of Cx47 GJ plaques per individual oligodendrocyte shows significant reduction in LPS compared to saline treated mice, most severely in KO T55I (h, j, l) (Student’s *t*-test, *:*p* < 0.05, **:*p* < 0.01, ***:*p* < 0.001). (TIF 9244 kb) Additional file 11: Figure S9. Disruption of Cx47 GJs in oligodendrocytes. Confocal microscopy images of cerebellar white matter sections from saline (a) and LPS (b) treated mice immunostained for Cx47 (red). Oligodendrocytes are green fluorescent because they transgenically express EGFP. Compared to saline control tissue with numerous Cx47 formed GJ plaques (open arrowheads) on the surface of oligodendrocytes (a), there is loss of plaques and increased intracellular immunoreactivity (arrow) in LPS tissue (b). Scale bar: 10 μm. (TIF 2639 kb) Additional file 12: Figure S10. Lack of oligodendrocyte apoptosis after LPS-induced neuroinflammation in Cx32 mutant mice. Images of fixed longitudinal sections of cerebellar white matter double stained with apoptosis marker caspase-3 (red) along with oligodendrocyte marker CC1 (green) and counter stained with DAPI (blue). Caspase-3 immunoreactivity does not increase in LPS-tissues (b, d, f) compared to saline controls (a, c, e) in any of the three genotypes, indicating lack of oligodendrocyte apoptosis up to 1 week after LPS-induced inflammation. Scale bar: 50 μm. (TIF 8299 kb) Additional file 13: Figure S11. LPS disrupts Cx43 and Cx47 GJs in spinal cord gray matter. a-f: Fixed longitudinal spinal cord gray matter immunostained for Cx43 (green) and Cx47 (red) and counterstained with nuclear marker DAPI (blue). Cx43 immunoreactivity is reduced in LPS-injected mice (b, d, f) of all genotypes compared to saline controls (a, c, e) as indicated. Scale bar in a-f: 10 μm. Counts of Cx43 GJs in all three genotypic groups confirms significant reduction in LPS injected compared to saline treated mice (g, i, k). Likewise, Cx47 GJ plaque numbers per area are also reduced in all genotypes after LPS injection (h, j, l) (Student’s *t*-test, *:*p* < 0.05, **:*p* < 0.01, ***:*p* < 0.001). (TIF 14930 kb) Additional file 14: Figure S12. LPS disrupts Cx43 and Cx47 GJ formation in the brainstem. a-f. Fixed coronal brainstem sections immunostained for Cx43 (green) and Cx47 (red) along with nuclear DAPI staining (blue). Cx43 immunoreactivity is reduced in the brainstem of LPS-injected mice (b, d, f) of all genotypes compared to their controls (a, c, e), associated with reduction of Cx47 GJ plaques. Scale bar: 10 μm. Quantification of total Cx43 GJ plaques confirms that LPS causes significant reduction of both Cx43 (g, i, k), as well as Cx47 formed GJs (h, j, l) in all three genotypic groups (Student’s *t*-test, *:*p* < 0.05, **:*p* < 0.01, ***:*p* < 0.001). (TIF 11587 kb) Additional file 15: Figure S13. Disruption of astrocyte and oligodendrocyte GJs in inflamed cerebellum. a-f: Fixed coronal cerebellar cortex sections including white matter (WM) surrounded by the granule cell layer (GCL) show double immunostaining with Cx43 (green), Cx47 (red) and nuclear DAPI staining (blue). Immunoreactivity of both Cx43 and Cx47 is reduced in LPS treated mice of all genotypes (b, d, f) compared to their saline controls (a, c, e) as indicated. Insets showing higher magnification of individual oligodendrocytes show reduction of GJ plaque formation by Cx43 and Cx47 at the cell bodies and proximal processes of oligodendrocytes with a weak diffuse cytoplasmic Cx47 immunoreactivity indicating intracellular diffusion (f). Scale bar: 50 μm. (TIF 21393 kb) Additional file 16: Figure S14. LPS does not induce astrocyte loss or astrogliosis in Cx32 KO or KO T55I mice. These are images of spinal cord white matter longitudinal sections immunostained with astrocytic marker GFAP (green) and astrocytic Cx43 (red). Cell nuclei are stained with DAPI (blue). When comparing saline to LPS treated WT (a, b), KO (c, d) and KO T55I (e, f) mice there is no apparent change in astrocyte immunoreactivity, while Cx43 appears to form fewer GJ plaques in LPS treated (b, d, f) compared to saline treated mice (a, c, e). Scale bar: 50 μm. (TIF 19981 kb) Additional file 17: Figure S15. Upregulation of ER-stress marker CHOP in oligodendrocytes of T55I KO mice treated with LPS. These are images of cerebellar white matter sections from saline (S) and LPS treated WT (a, b), Cx32 KO (c, d) and KO T55I (e, f) mice, as indicated, immunostained with oligodendrocyte marker CC1 (green) and ER-stress response marker CHOP (red). Cell nuclei are stained with DAPI (blue). Details of oligodendrocytes are shown in insets and separate channels. CHOP immunoreactivity is detectable in oligodendrocytes of KO T55I mice treated with LPS (open arrowheads in f) but not in the other treatment groups. Scale bar: 10 μm. (TIF 13422 kb)
