# Supplementary figures and images for: Systemic inflammation disrupts oligodendrocyte gap junctions and induces ER stress in a model of CNS manifestations of X-linked Charcot-Marie-Tooth disease
Source: Acta Neuropathol Commun. 2016 Sep 1;4(1):95. doi: 10.1186/s40478-016-0369-5 (PMC5009701; doi:10.1186/s40478-016-0369-5)

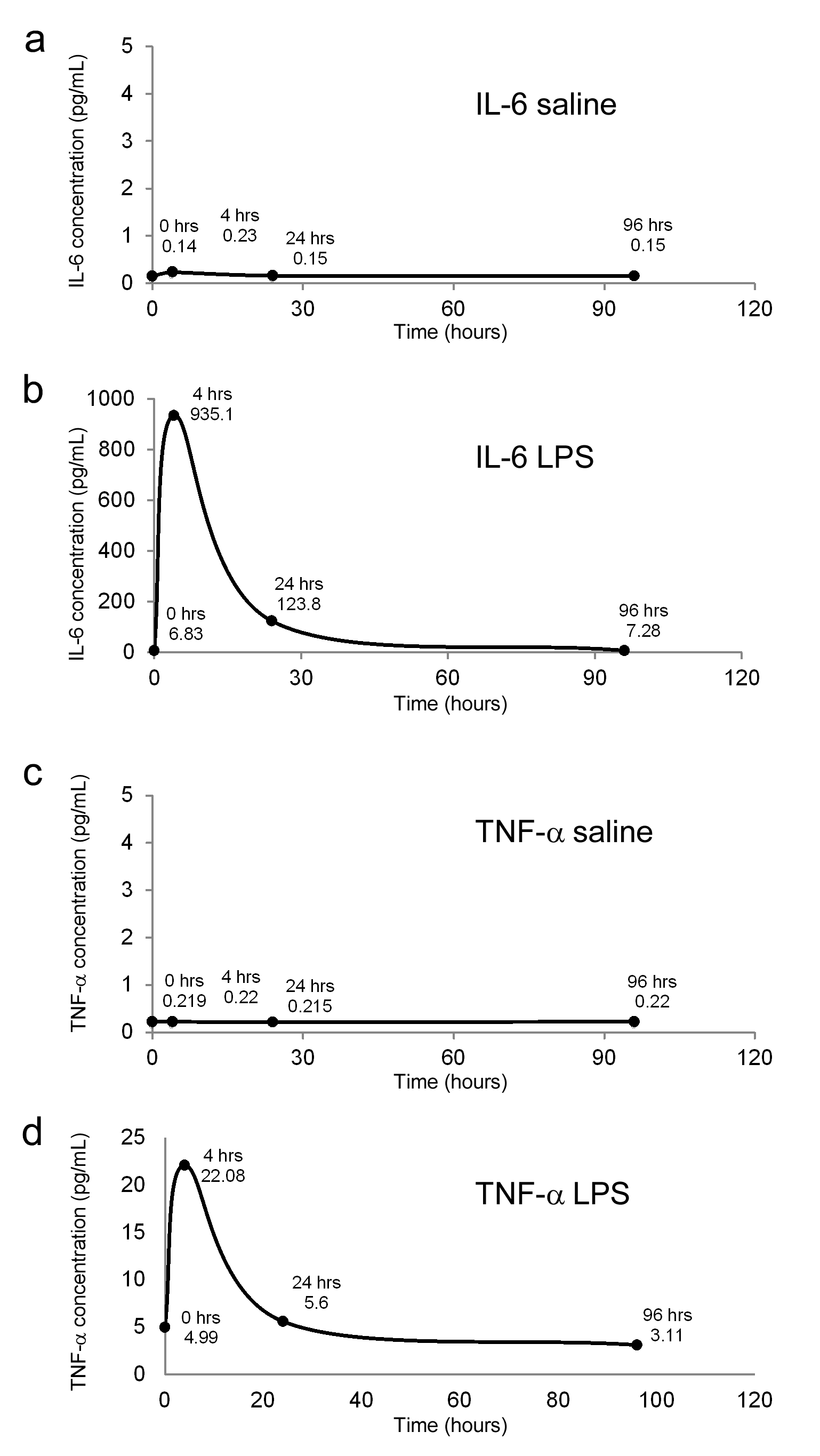

Supplement: Additional file 1: Figure S1. — 1 LPS induces IL-6 and TNF-α increase in Cx32 KO mice. a-b: Graphs representing concentration course of IL-6 before (0 h) as well as 4, 24, and 96 h after saline (a) or LPS (b) injection as measured at 450 nm with ELISA. Saline did not cause any significant systemic inflammation (baseline inflammation: 0.14 pg/mL, 4 h after injection: 0.23 pg/mL), whereas a marked increase 4 h after LPS injection (935.1 pg/mL) was found. c-d: Levels of TNF-α before (0 h) as well as after (4, 24, 96 h) saline (c) and LPS (d) injection; saline did not cause any inflammatory response (baseline: 0.21 pg/mL, at 4 h: 0.22 pg/mL) whereas LPS injection caused a marked TNF-α increase (4 h after injection: 22.08 pg/mL). (TIF 12301 kb) [file 40478_2016_369_MOESM1_ESM.tif]

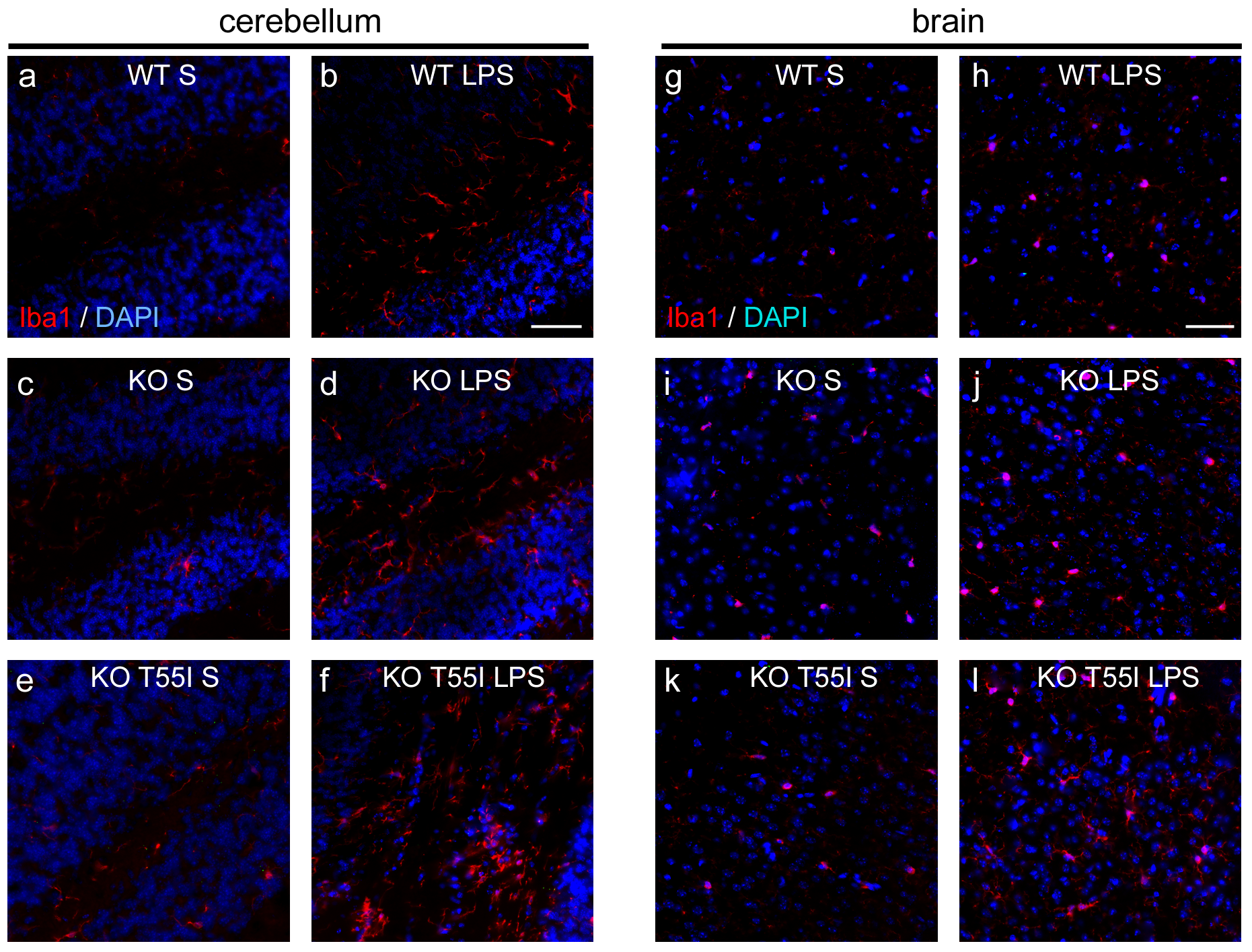

Supplement: Additional file 2: Figure S2. — LPS causes diffuse CNS inflammation in the cerebellum and cerebral cortex. Images of fixed coronal sections of cerebellum (a-f) and cerebral cortex (g-l), as indicated, immunostained for microglial marker Iba1 (red). Cell nuclei are counterstained with DAPI (blue). Microglia are diffusely activated in both CNS areas and in all genotypes in LPS (b, d, f, h, j, l) compared to saline (a, c, e, g, i, k) injected mice. Scale bars: 50 μm. (TIF 7715 kb) [file 40478_2016_369_MOESM2_ESM.tif]

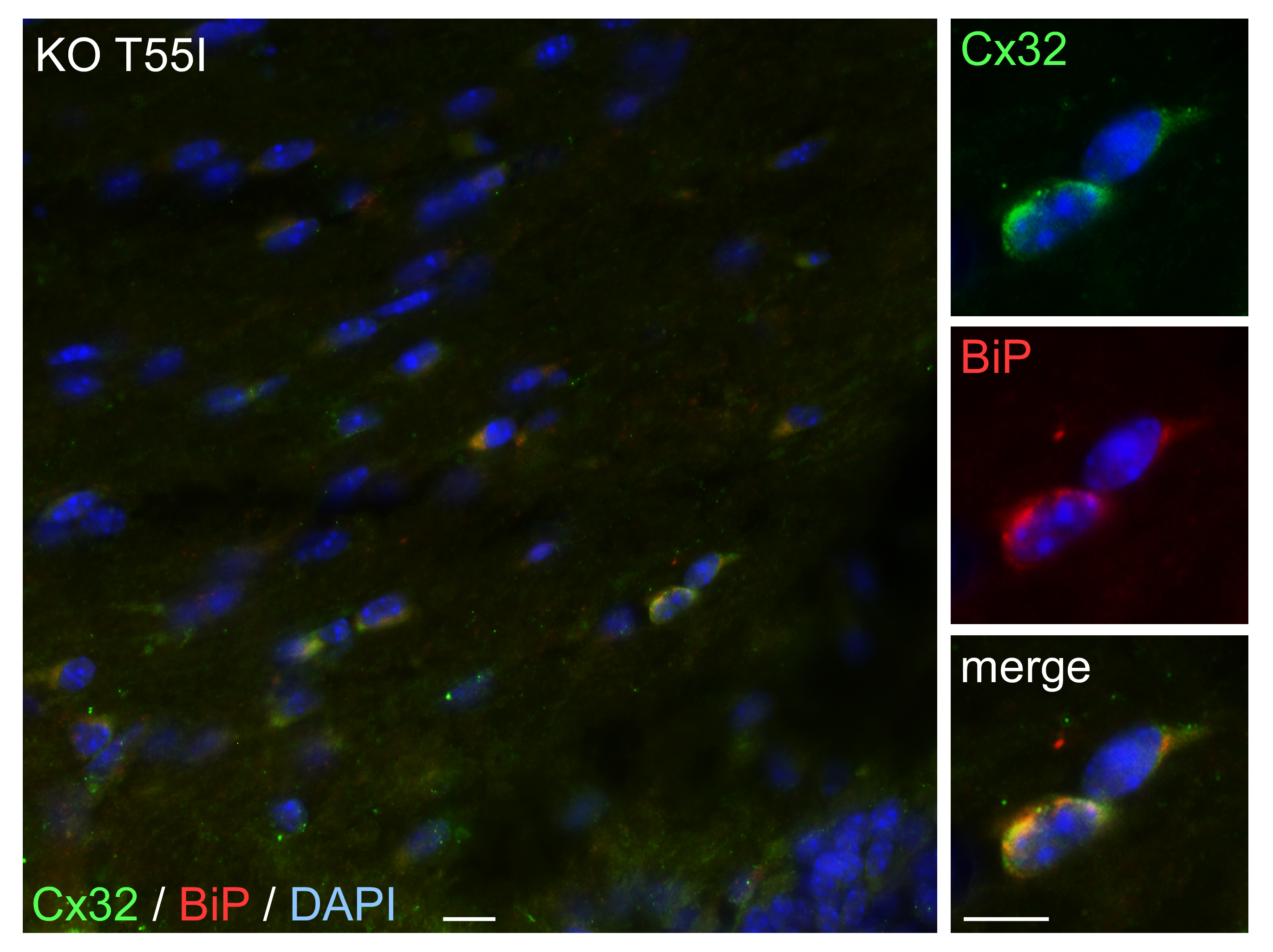

Supplement: Additional file 4: Figure S3. — Localization of the T55I Cx32 mutant in the endoplasmic reticulum in oligodendrocytes. This is an image of spinal cord white matter longitudinal sections from a Cx32 KO T55I mutant mouse (saline control) double labeled with antibodies to Cx32 (green) and ER marker BiP (red). Cell nuclei are stained with DAPI (blue). Overview image is shown on the left and higher magnification images of cells in separate channels are shown on the right. The T55I mutant is retained intracellularly in oligodendrocytes and colocalizes in the perinuclear cytoplasm with the ER marker, indicating retention in the ER. No GJ-like plaques are formed by the mutant on the cell membrane. Scale bar: 10 μm. (TIF 16462 kb) [file 40478_2016_369_MOESM4_ESM.tif]

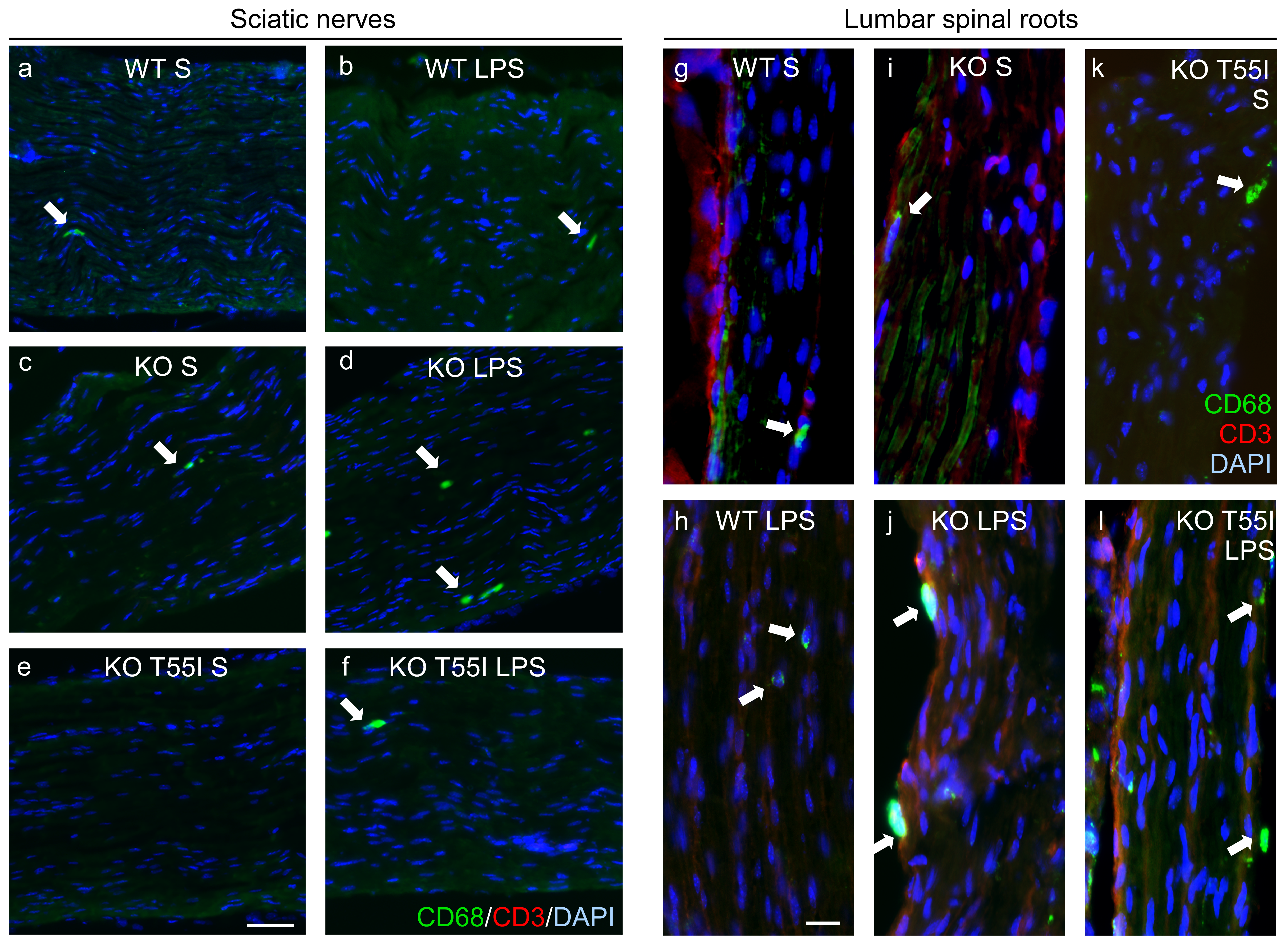

Supplement: Additional file 5: Figure S4. — LPS injection does not cause inflammatory changes in the peripheral nervous system. Fixed longitudinal sections of sciatic nerves (a-f) and lumbar spinal roots (g-l) from LPS-injected and saline control mice from all three genotypes as indicated, immunostained with T-cell marker CD3 (red) and macrophage marker CD68 (green). Cell nuclei are stained with DAPI (blue). There is no difference in inflammatory cell immunoreactivity between treatment groups in any of the genotypes. No CD3+ cells are seen, whereas 1–2 CD68+ macrophages are present in all tissues regardless of the genotype and treatment condition. Scale bars in a-f: 30 μm; in g-l: 20 μm. (TIF 50275 kb) [file 40478_2016_369_MOESM5_ESM.tif]

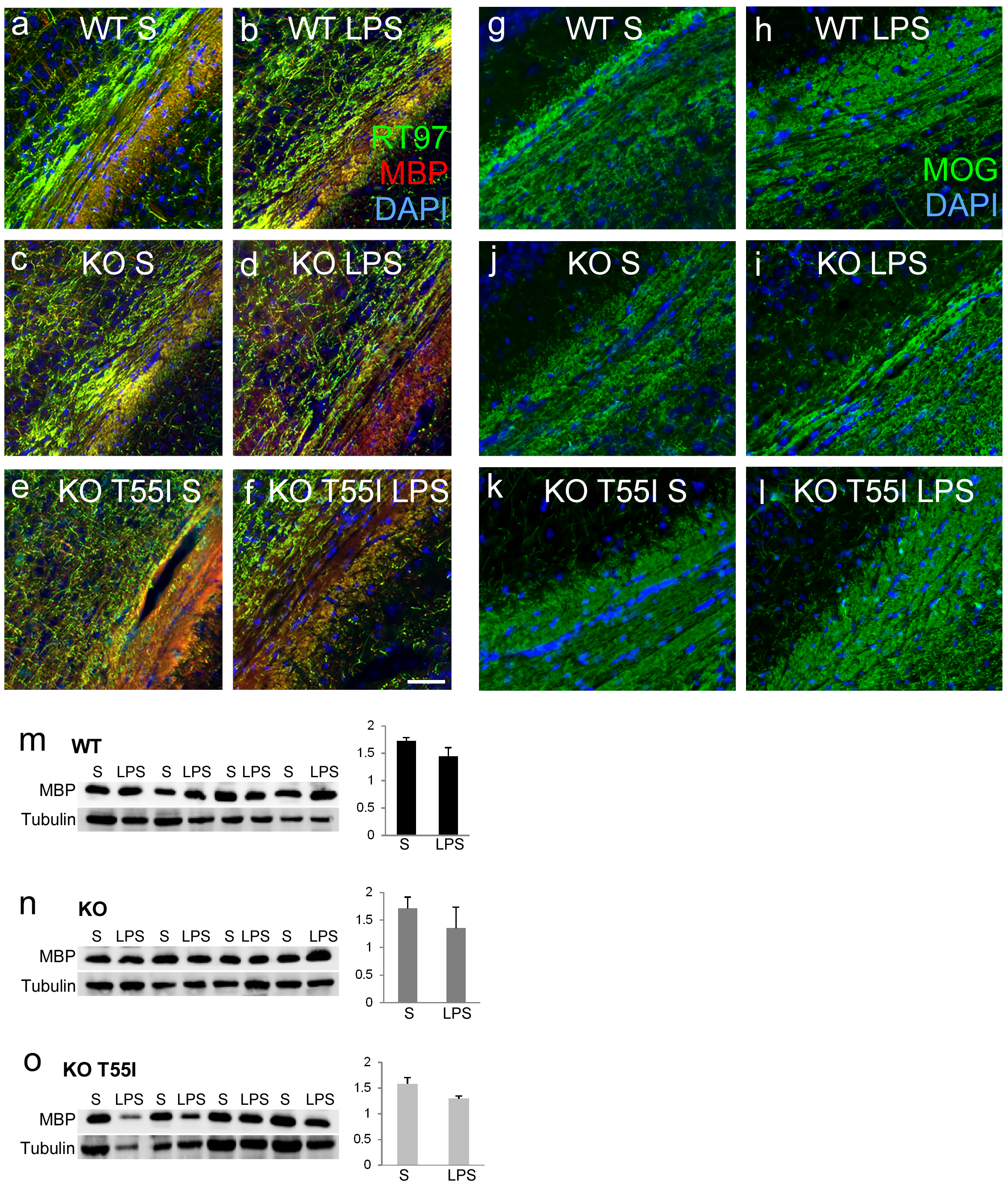

Supplement: Additional file 7: Figure S5. — Lack of CNS demyelination in LPS-injected Cx32 mutant mice. a-l: Images of brain sections at the level of the corpus callosum immunostained with axonal marker RT97 (green) in combination with myelin marker MBP (red) (a-f) or with myelin marker MOG (g-l) and DAPI nuclear staining (blue). There is no apparent alteration of myelin immunoreactivity in the brains of LPS-injected mice (b, d, f, h, I, l) compared to saline controls (a, c, e, g, j, k) from all three genotypes, as indicated. Scale bar: 50 μm. m-o: Immunoblot analysis of MBP levels in brainstem tissue lysates show no significant change induced by LPS injection (LPS) compared to saline-injected mice (S) in WT (m), Cx32 KO (n) or KO T55I (o) groups as indicated. All blots were re-probed for tubulin to demonstrate the loading, and quantification of normalized MBP band intensity is shown next to each blot. (TIF 19222 kb) [file 40478_2016_369_MOESM7_ESM.tif]

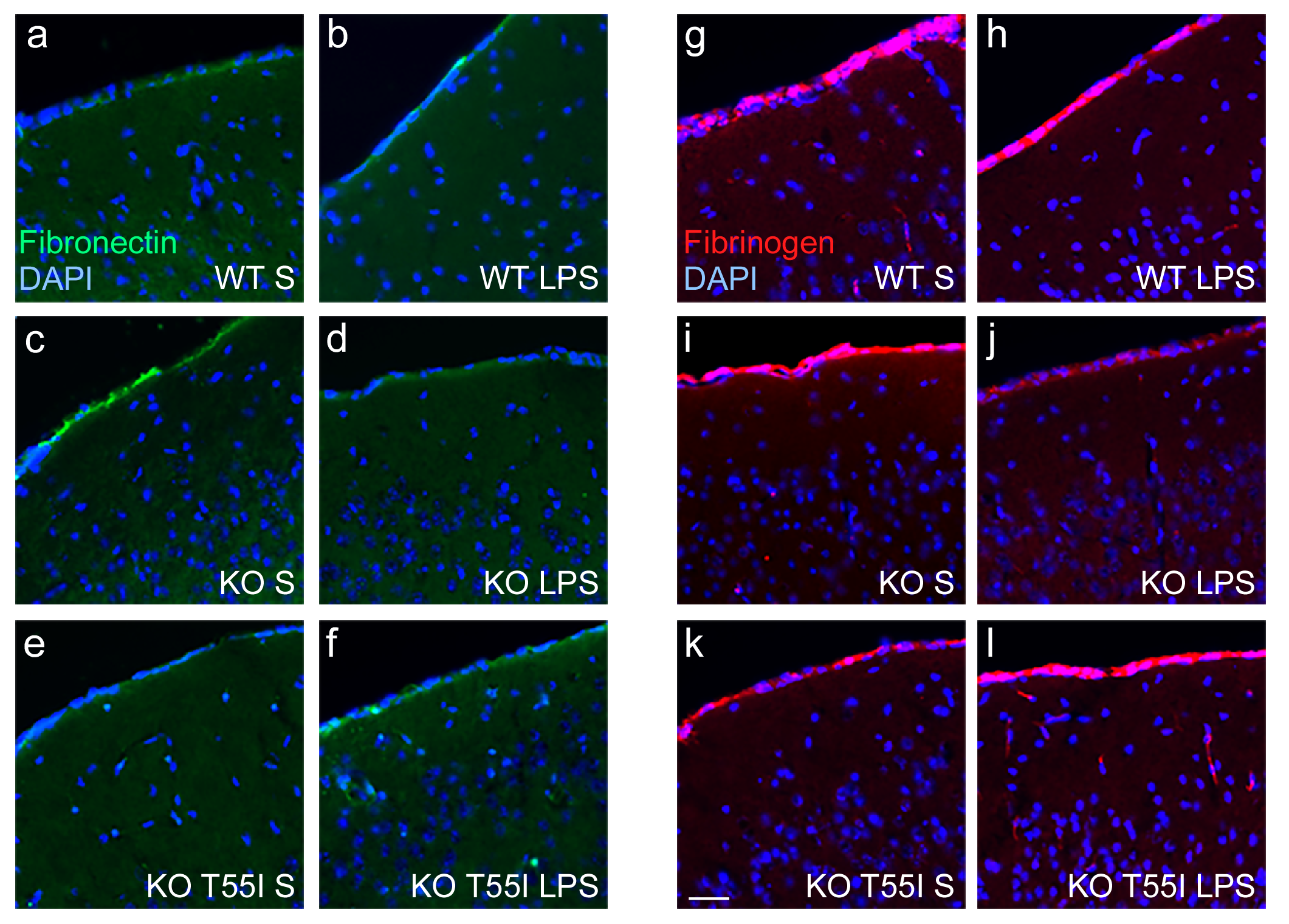

Supplement: Additional file 8: Figure S6. — LPS induced neuroinflammation does not cause blood brain barrier (BBB) disruption in Cx32 mutant mice. These are images of fixed coronal brain sections, immunostained with blood brain barrier markers fibronectin (green) (a-f) or fibrinogen (red) (g-l) and counter stained with DAPI (blue). There is no apparent disturbance of BBB integrity in the brain of LPS treated (b, d, f, h, j, l) compared to saline treated (a, c, e, g, i, k) mice from any of the three genotypes, as indicated. Linear fibrinogen immunoreactivity in brain parenchyma represents small blood vessels. Scale bar: 50 μm. (TIF 9451 kb) [file 40478_2016_369_MOESM8_ESM.tif]

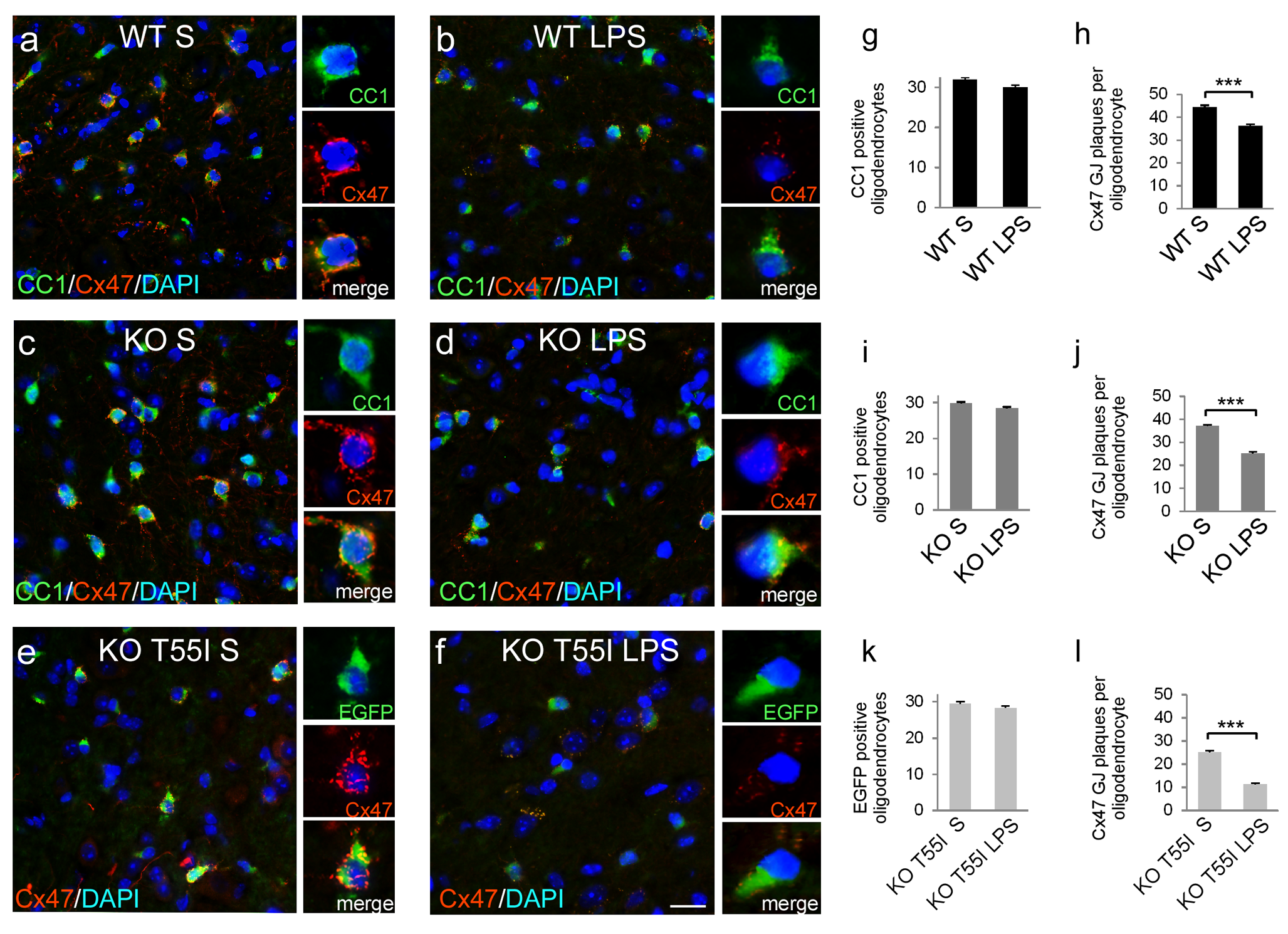

Supplement: Additional file 9: Figure S7. — LPS disrupts oligodendrocyte GJs formed by Cx47 in spinal cord gray matter. a-f: Fixed longitudinal spinal cord gray matter sections immunostained with oligodendrocyte marker CC1 (green) and Cx47 (red) and counterstained with DAPI (blue). In KO T55I tissues all oligodendrocytes transgenically express EGFP and were not stained with CC1. Cx47 immunoreactivity and GJ plaque formation at oligodendrocyte cell bodies and proximal processes is reduced in LPS-treated mice (b, d, f) of all genotypes compared to their saline controls (a, c, e). Scale bar: 20 μm. Counts of Cx47 GJ plaques per individual oligodendrocyte shows a significant reduction in LPS compared to saline treated mice (h, j, l), whereas the number of CC1/EGFP-positive oligodendrocytes per genotype as indicated shows no significant reduction in LPS compared to saline treated mice (Student’s t-test, *:p < 0.05, **:p < 0.01, ***:p < 0.001). (TIF 8996 kb) [file 40478_2016_369_MOESM9_ESM.tif]

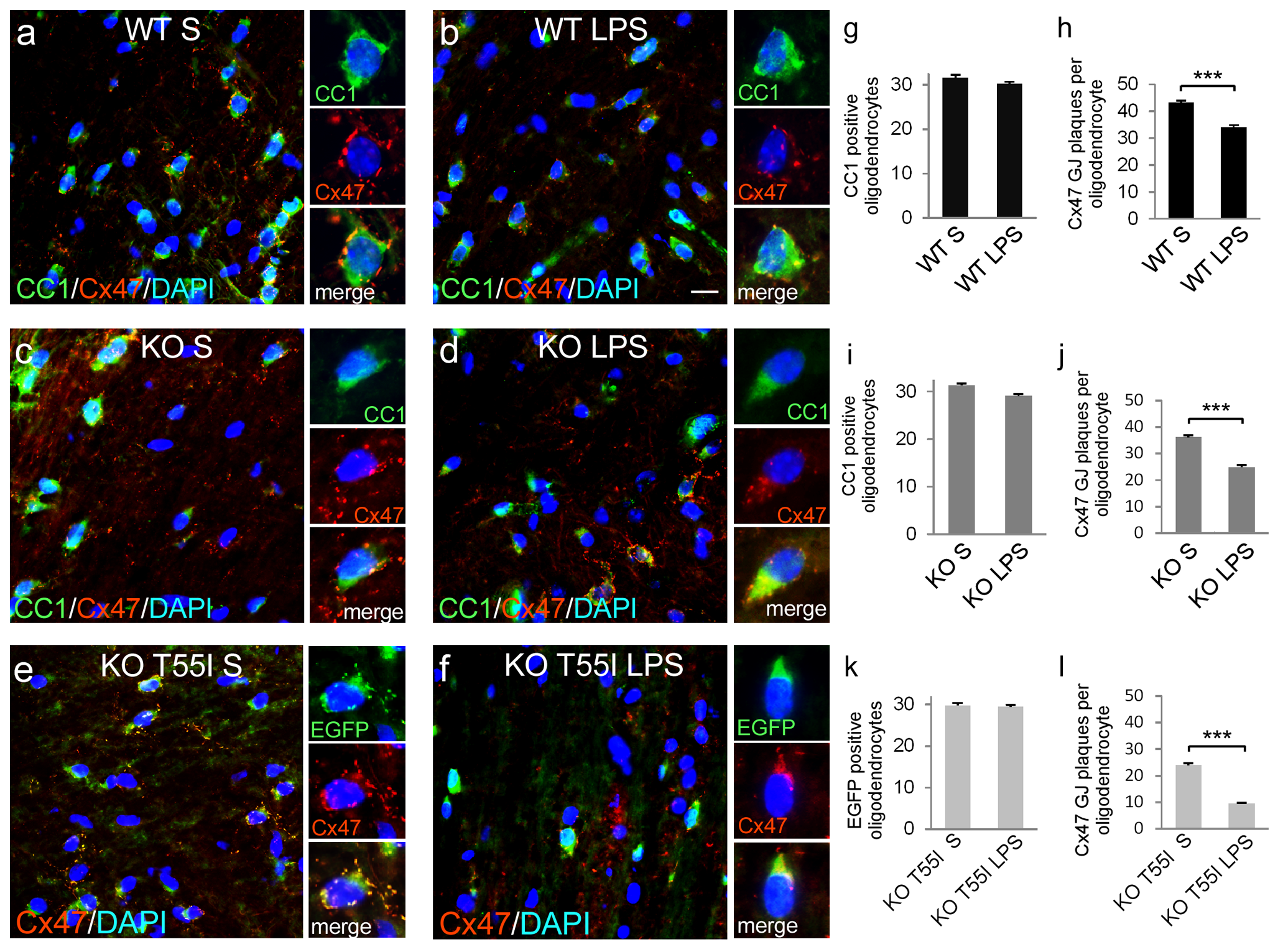

Supplement: Additional file 10: Figure S8. — LPS disrupts oligodendrocyte Cx47 GJs in spinal cord WM. a-f: Fixed longitudinal spinal cord WM sections immunostained for CC1 (green) and Cx47 (red) and counterstained with DAPI (blue). In KO T55I tissues all oligodendrocytes transgenically express EGFP and were not stained with CC1. Cx47 immunoreactivity and GJ plaque formation at oligodendrocyte cell bodies and proximal processes is reduced in LPS treated mice of all genotypes (b, d, f) compared to their saline controls (a, c, e), with increased cytoplasmic immunoreactivity insets in d, f). Scale bar: 10 μm. Quantification of CC1/EGFP-positive oligodendrocytes per genotype as indicated (g, i, k) shows no significant reduction in LPS treated compared to saline groups, whereas quantification of Cx47 GJ plaques per individual oligodendrocyte shows significant reduction in LPS compared to saline treated mice, most severely in KO T55I (h, j, l) (Student’s t-test, *:p < 0.05, **:p < 0.01, ***:p < 0.001). (TIF 9244 kb) [file 40478_2016_369_MOESM10_ESM.tif]

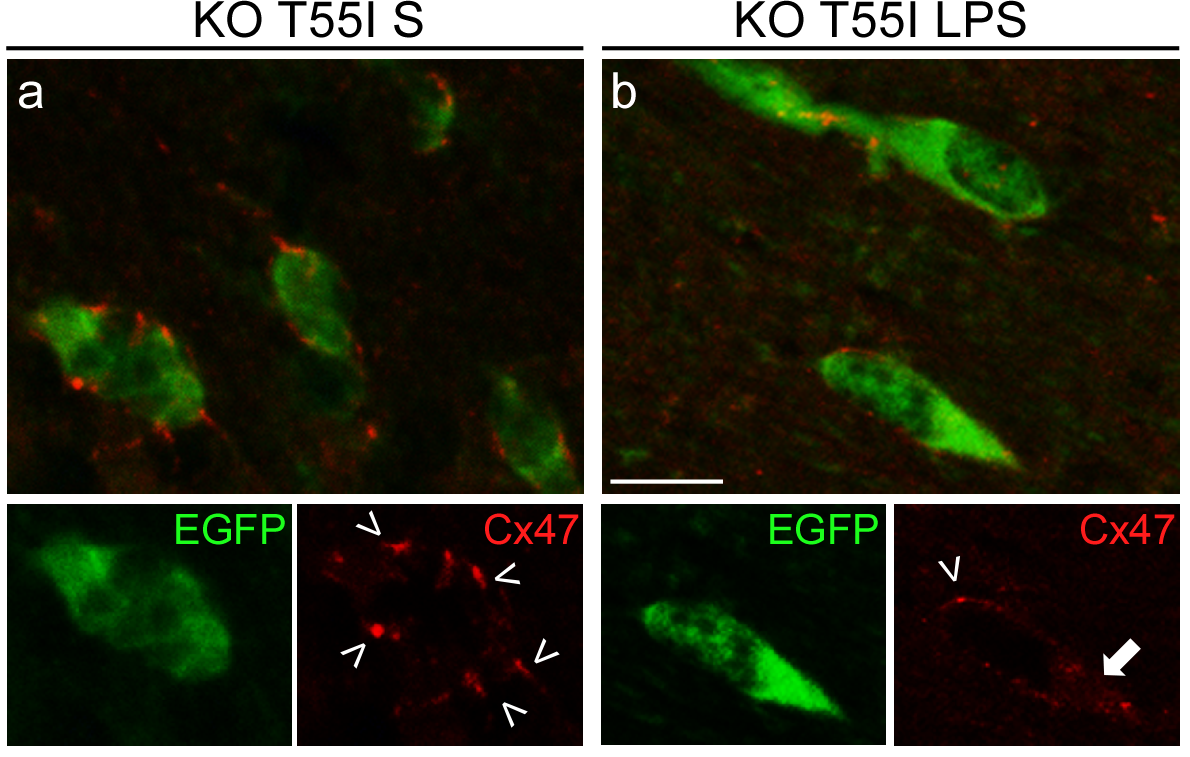

Supplement: Additional file 11: Figure S9. — Disruption of Cx47 GJs in oligodendrocytes. Confocal microscopy images of cerebellar white matter sections from saline (a) and LPS (b) treated mice immunostained for Cx47 (red). Oligodendrocytes are green fluorescent because they transgenically express EGFP. Compared to saline control tissue with numerous Cx47 formed GJ plaques (open arrowheads) on the surface of oligodendrocytes (a), there is loss of plaques and increased intracellular immunoreactivity (arrow) in LPS tissue (b). Scale bar: 10 μm. (TIF 2639 kb) [file 40478_2016_369_MOESM11_ESM.tif]

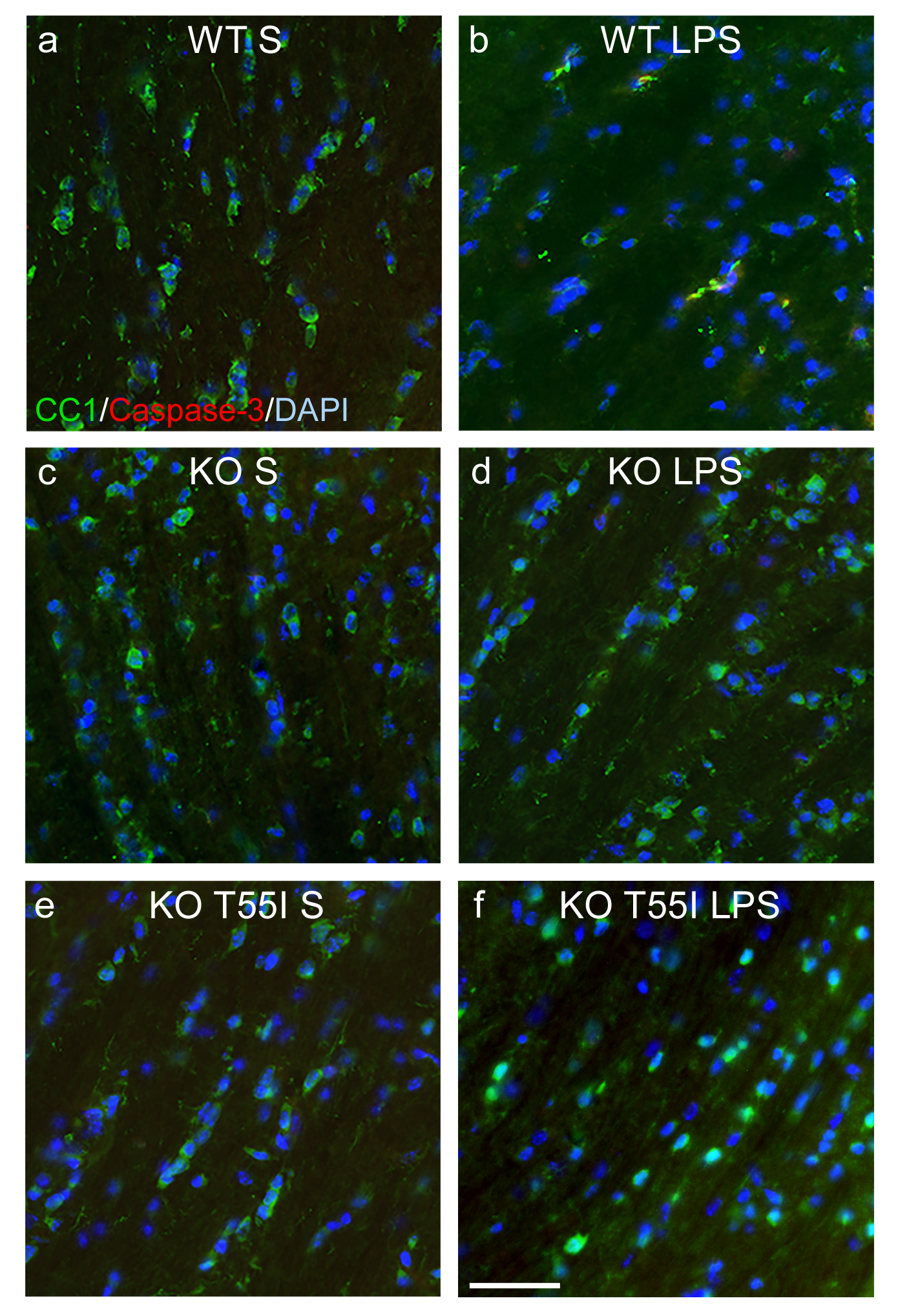

Supplement: Additional file 12: Figure S10. — Lack of oligodendrocyte apoptosis after LPS-induced neuroinflammation in Cx32 mutant mice. Images of fixed longitudinal sections of cerebellar white matter double stained with apoptosis marker caspase-3 (red) along with oligodendrocyte marker CC1 (green) and counter stained with DAPI (blue). Caspase-3 immunoreactivity does not increase in LPS-tissues (b, d, f) compared to saline controls (a, c, e) in any of the three genotypes, indicating lack of oligodendrocyte apoptosis up to 1 week after LPS-induced inflammation. Scale bar: 50 μm. (TIF 8299 kb) [file 40478_2016_369_MOESM12_ESM.tif]

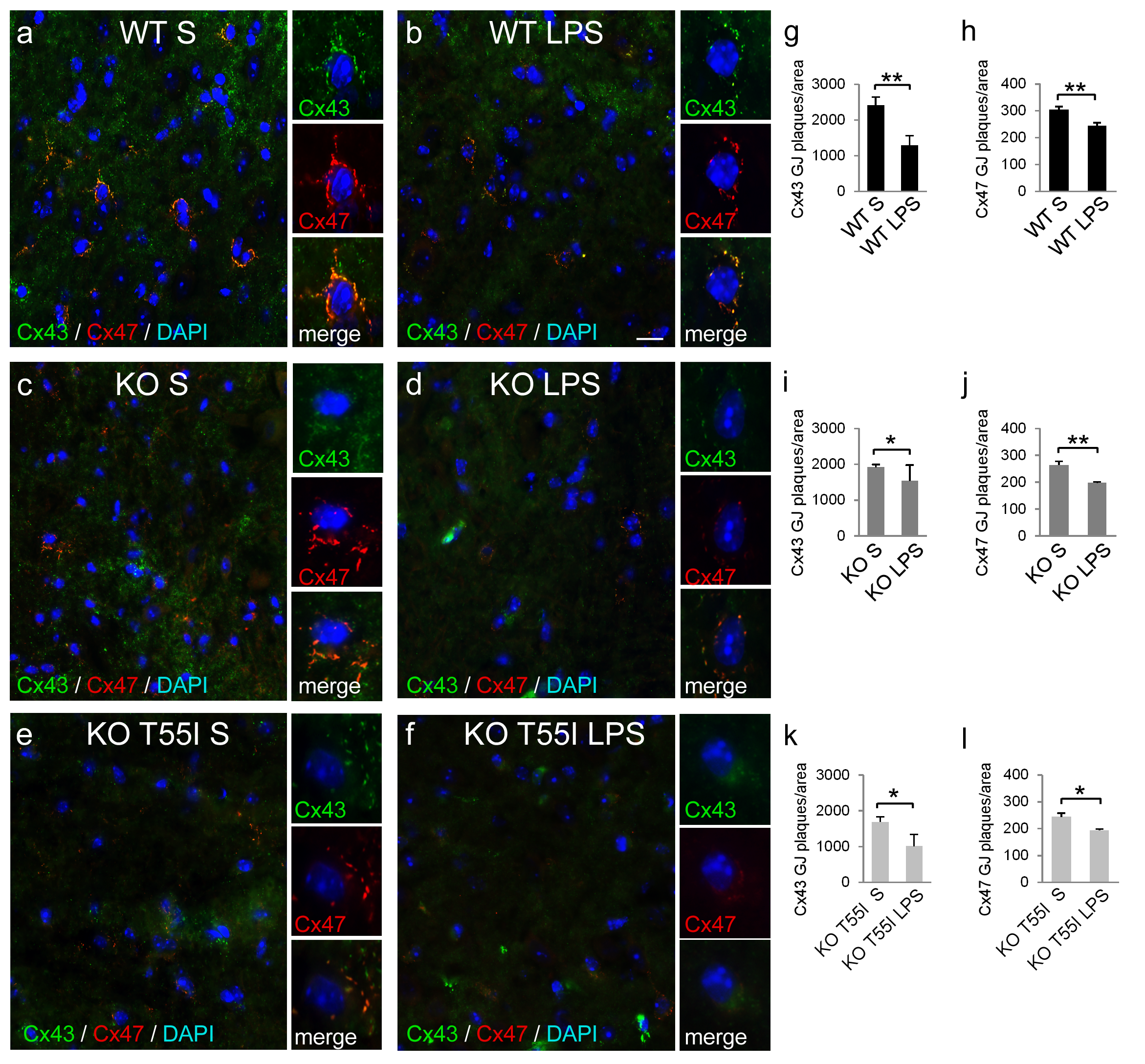

Supplement: Additional file 13: Figure S11. — LPS disrupts Cx43 and Cx47 GJs in spinal cord gray matter. a-f: Fixed longitudinal spinal cord gray matter immunostained for Cx43 (green) and Cx47 (red) and counterstained with nuclear marker DAPI (blue). Cx43 immunoreactivity is reduced in LPS-injected mice (b, d, f) of all genotypes compared to saline controls (a, c, e) as indicated. Scale bar in a-f: 10 μm. Counts of Cx43 GJs in all three genotypic groups confirms significant reduction in LPS injected compared to saline treated mice (g, i, k). Likewise, Cx47 GJ plaque numbers per area are also reduced in all genotypes after LPS injection (h, j, l) (Student’s t-test, *:p < 0.05, **:p < 0.01, ***:p < 0.001). (TIF 14930 kb) [file 40478_2016_369_MOESM13_ESM.tif]

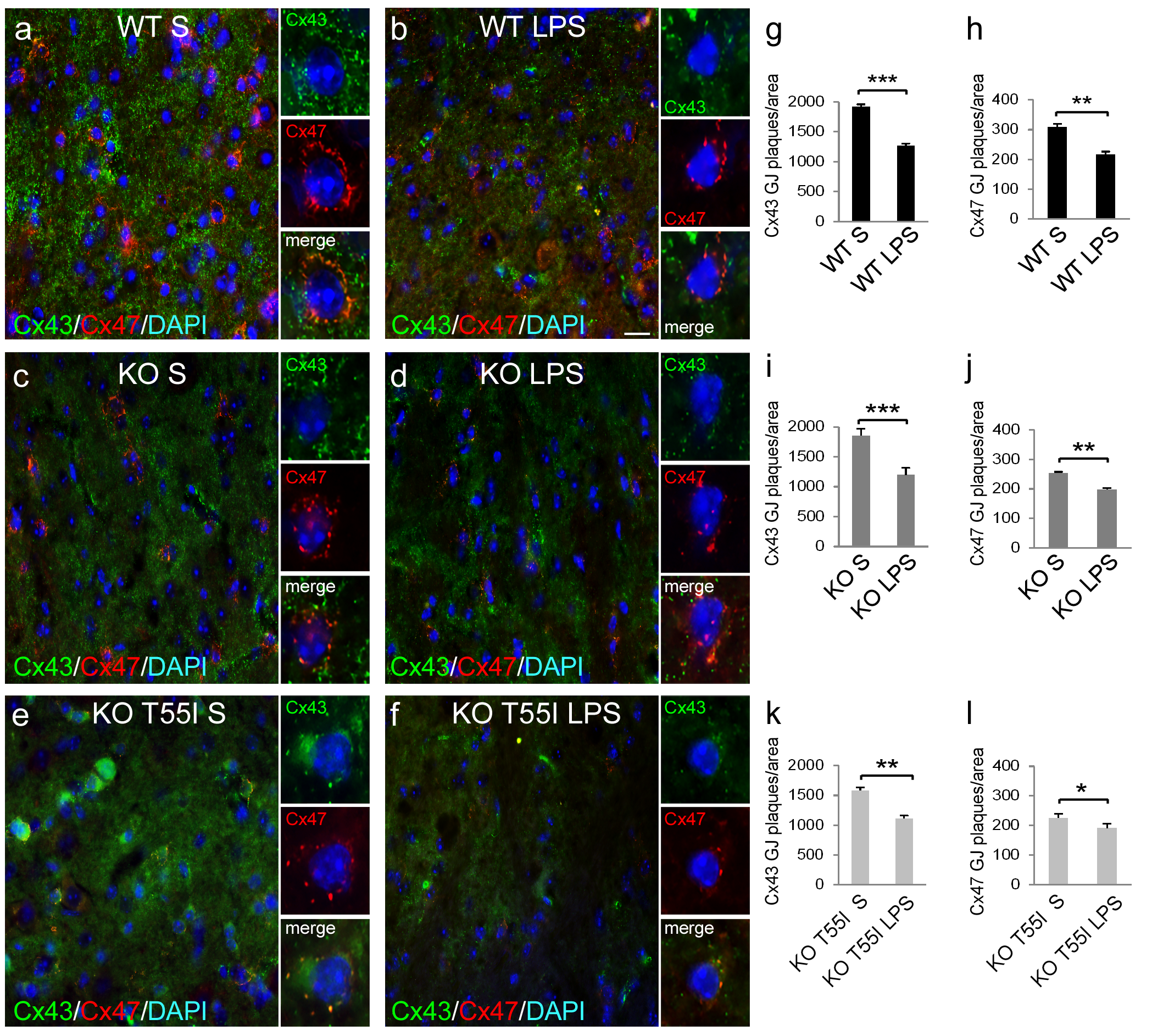

Supplement: Additional file 14: Figure S12. — LPS disrupts Cx43 and Cx47 GJ formation in the brainstem. a-f. Fixed coronal brainstem sections immunostained for Cx43 (green) and Cx47 (red) along with nuclear DAPI staining (blue). Cx43 immunoreactivity is reduced in the brainstem of LPS-injected mice (b, d, f) of all genotypes compared to their controls (a, c, e), associated with reduction of Cx47 GJ plaques. Scale bar: 10 μm. Quantification of total Cx43 GJ plaques confirms that LPS causes significant reduction of both Cx43 (g, i, k), as well as Cx47 formed GJs (h, j, l) in all three genotypic groups (Student’s t-test, *:p < 0.05, **:p < 0.01, ***:p < 0.001). (TIF 11587 kb) [file 40478_2016_369_MOESM14_ESM.tif]

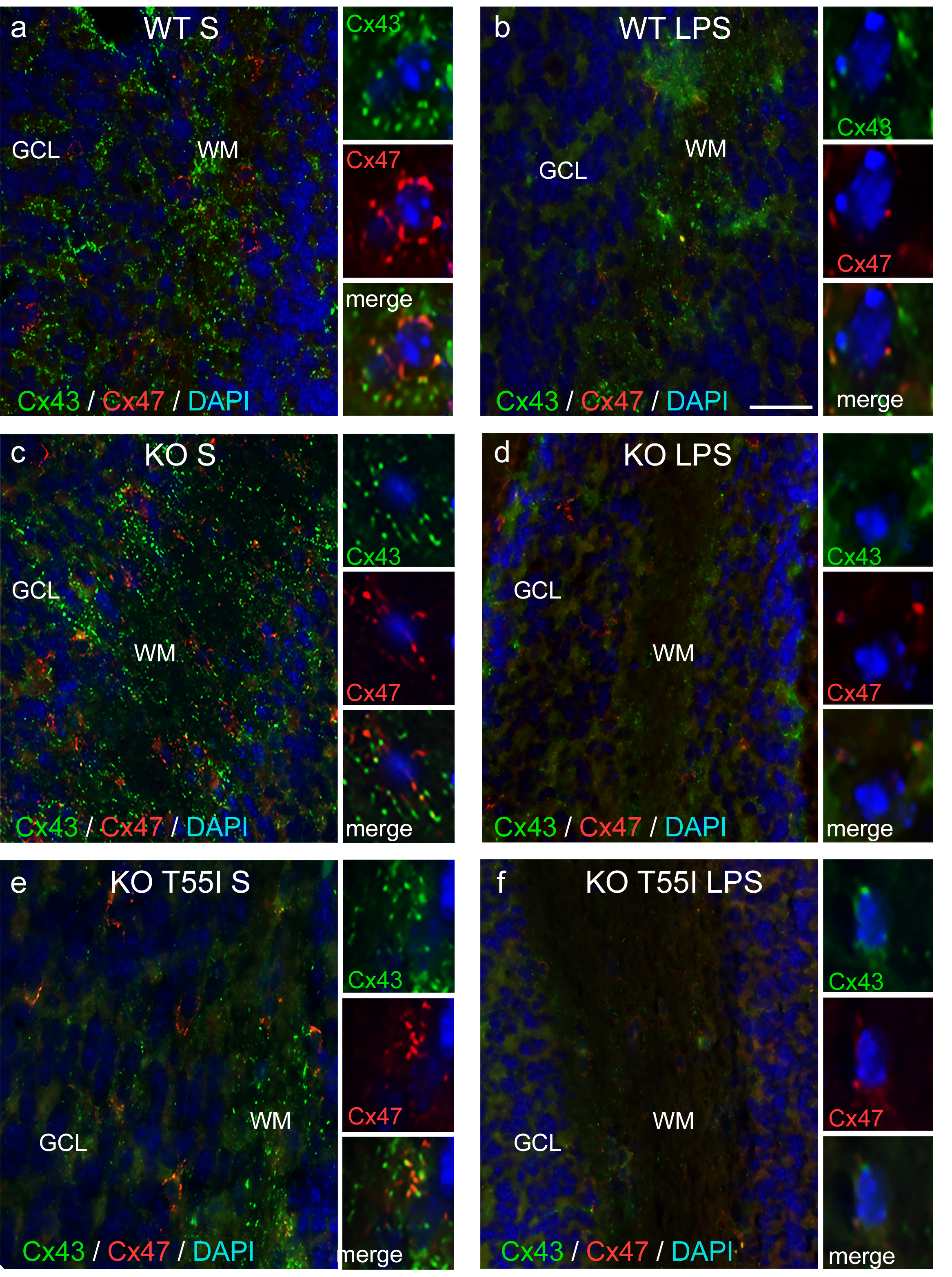

Supplement: Additional file 15: Figure S13. — Disruption of astrocyte and oligodendrocyte GJs in inflamed cerebellum. a-f: Fixed coronal cerebellar cortex sections including white matter (WM) surrounded by the granule cell layer (GCL) show double immunostaining with Cx43 (green), Cx47 (red) and nuclear DAPI staining (blue). Immunoreactivity of both Cx43 and Cx47 is reduced in LPS treated mice of all genotypes (b, d, f) compared to their saline controls (a, c, e) as indicated. Insets showing higher magnification of individual oligodendrocytes show reduction of GJ plaque formation by Cx43 and Cx47 at the cell bodies and proximal processes of oligodendrocytes with a weak diffuse cytoplasmic Cx47 immunoreactivity indicating intracellular diffusion (f). Scale bar: 50 μm. (TIF 21393 kb) [file 40478_2016_369_MOESM15_ESM.tif]

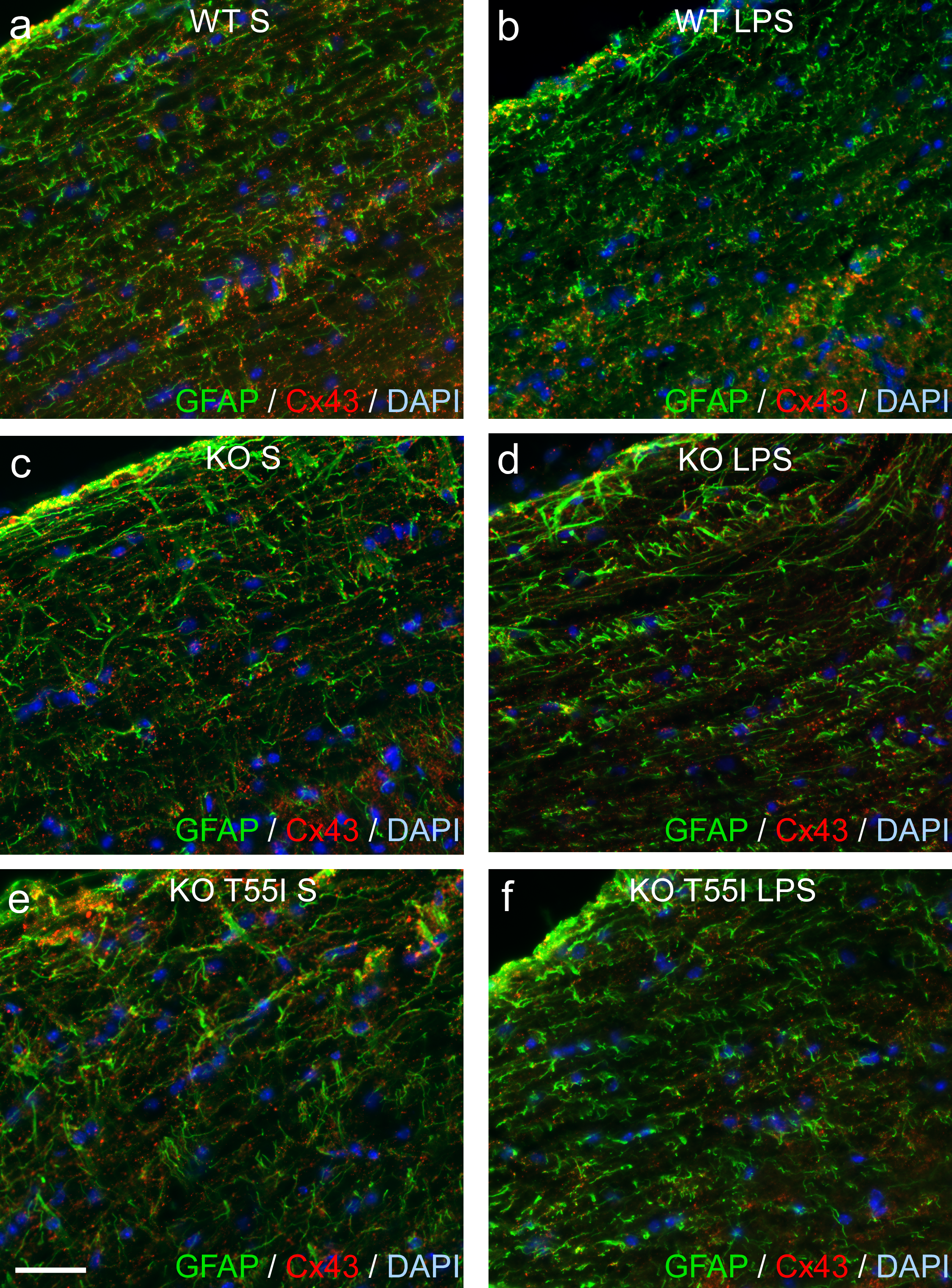

Supplement: Additional file 16: Figure S14. — LPS does not induce astrocyte loss or astrogliosis in Cx32 KO or KO T55I mice. These are images of spinal cord white matter longitudinal sections immunostained with astrocytic marker GFAP (green) and astrocytic Cx43 (red). Cell nuclei are stained with DAPI (blue). When comparing saline to LPS treated WT (a, b), KO (c, d) and KO T55I (e, f) mice there is no apparent change in astrocyte immunoreactivity, while Cx43 appears to form fewer GJ plaques in LPS treated (b, d, f) compared to saline treated mice (a, c, e). Scale bar: 50 μm. (TIF 19981 kb) [file 40478_2016_369_MOESM16_ESM.tif]

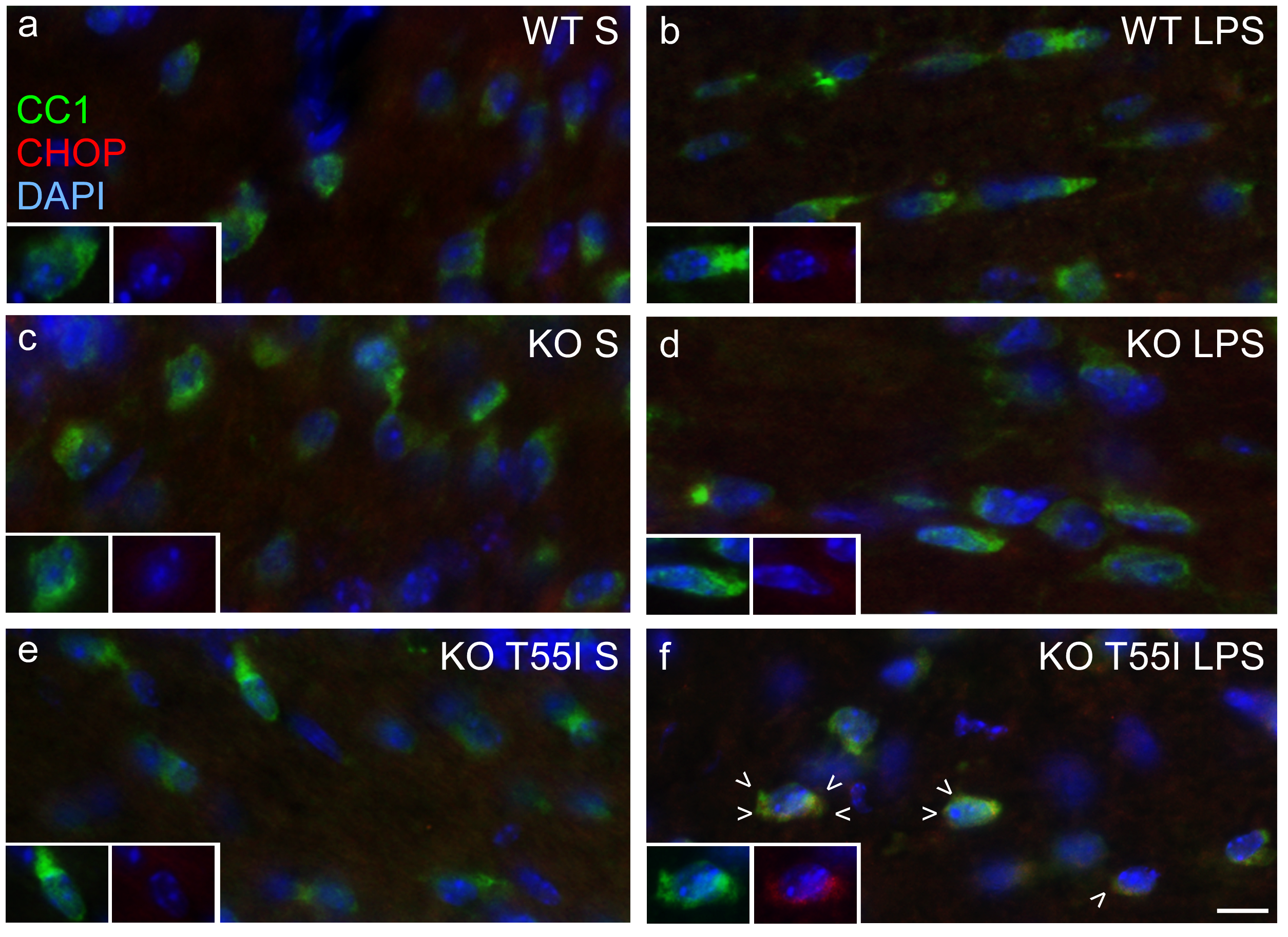

Supplement: Additional file 17: Figure S15. — Upregulation of ER-stress marker CHOP in oligodendrocytes of T55I KO mice treated with LPS. These are images of cerebellar white matter sections from saline (S) and LPS treated WT (a, b), Cx32 KO (c, d) and KO T55I (e, f) mice, as indicated, immunostained with oligodendrocyte marker CC1 (green) and ER-stress response marker CHOP (red). Cell nuclei are stained with DAPI (blue). Details of oligodendrocytes are shown in insets and separate channels. CHOP immunoreactivity is detectable in oligodendrocytes of KO T55I mice treated with LPS (open arrowheads in f) but not in the other treatment groups. Scale bar: 10 μm. (TIF 13422 kb) [file 40478_2016_369_MOESM17_ESM.tif]
