# Supplementary material for: Systemic inflammation disrupts oligodendrocyte gap junctions and induces ER stress in a model of CNS manifestations of X-linked Charcot-Marie-Tooth disease
Source: Acta Neuropathol Commun. 2016 Sep 1;4(1):95. doi: 10.1186/s40478-016-0369-5 (PMC5009701; doi:10.1186/s40478-016-0369-5)
Supplement: Additional file 3: Table S1. — Quantification of Iba1 immunoreactivity in different CNS areas of saline and LPS treated WT, Cx32 KO (KO) and T55I KO mice. (DOCX 22 kb) [file 40478_2016_369_MOESM3_ESM.docx]

**Additional file 3: Table S1:** **Quantification of Iba1 immunoreactivity in different CNS areas of saline and LPS treated WT, Cx32 KO (KO) and T55I KO mice.**

| **Genotype**  **and area** | **Saline** | **LPS** | **Saline vs. LPS** | **Comparing genotypes at baseline** | **Comparing genotypes after LPS** |
| --- | --- | --- | --- | --- | --- |
| **SPINAL CORD** |  |  |  |  |  |
| WT | 0.68 ± 0.16 | 2.22 ± 0.46 | p<0.001 | p<0.001 (KO) | p<0.001 (KO) |
| KO | 1.7 ± 0.26 | 3.21 ± 0.38 | p<0.001 | p=0.007 (KO T55I) | p<0.001 (KO T55I) |
| KO T55I | 1.88 ± 0.23 | 4.03 ± 0.43 | p<0.001 | p<0.001 (WT) | p<0.001 (WT) |
|  |  |  |  |  |  |
| **BRAINSTEM** |  |  |  |  |  |
| WT | 0.87 ± 0.13 | 2.76 ± 0.54 | p<0.001 | p<0.001 (KO) | p<0.001 (KO) |
| KO | 1.77 ± 0.17 | 4.09 ± 0.27 | p<0.001 | p=0.01 (KO T55I) | p<0.001 (KO T55I) |
| KO T55I | 2.04 ± 0.23 | 4.74 ± 0.31 | p<0.001 | p<0.001 (WT) | p<0.001 (WT) |
|  |  |  |  |  |  |
| **CEREBELLUM** |  |  |  |  |  |
| WT | 1 ± 0.24 | 2.07 ± 0.27 | p<0.001 | p<0.001 (KO) | p<0.001 (KO) |
| KO | 1.94 ± 0.23 | 4.02 ± 0.33 | p<0.001 | n.s. (KO T55I) | p<0.001 (KO T55I) |
| KO T55I | 2.03 ± 0.36 | 4.82 ± 0.39 | p<0.001 | p<0.001 (WT) | p<0.001 (WT) |
|  |  |  |  |  |  |
| **BRAIN** |  |  |  |  |  |
| WT | 0.84 ± 0.09 | 2.34 ± 0.28 | p=0.03 | p<0.001 (KO) | p=0.04 (KO) |
| KO | 1.68 ± 0.05 | 3.83 ± 0.67 | p=0.02 | n.s. (KO T55I) | p=0.004 (KO T55I) |
| KO T55I | 1.89 ± 0.12 | 6.09 ± 0.79 | p<0.001 | p<0.001 (WT) | p<0.001 (WT) |

The average ± SD results for each genotype group are shown. *p* values obtained with the Student’s t-test and significant results *after Bonferroni correction* for multiple comparisons are shown (the group with which comparison was made is indicated in parentheses). n.s.: non-significant.
