# Supplementary material for: Systemic inflammation disrupts oligodendrocyte gap junctions and induces ER stress in a model of CNS manifestations of X-linked Charcot-Marie-Tooth disease
Source: Acta Neuropathol Commun. 2016 Sep 1;4(1):95. doi: 10.1186/s40478-016-0369-5 (PMC5009701; doi:10.1186/s40478-016-0369-5)
Supplement: Additional file 6: Table S2. — Results of behavioral testing in saline control and LPS treated WT, Cx32 KO (KO) and T55I KO mice. (DOCX 22 kb) [file 40478_2016_369_MOESM6_ESM.docx]

**Additional file 6: Table S2:** **Results of behavioral testing in saline control and LPS treated WT, Cx32 KO (KO) and T55I KO mice.**

|  | | | | | |
| --- | --- | --- | --- | --- | --- |
| **Genotype** | **Saline** | **LPS** | **Saline vs. LPS** | **Comparing genotypes at baseline** | **Comparing genotypes after LPS** |
| **Rotarod 12 RPM (Time-seconds)** | | |  |  |  |
| WT | 897.45 ± 34.46 | 431.61 ± 57.41 | p<0.001 | p=0.001 (KO) | p=0.01 (KO) |
| KO | 769 ± 94.39 | 283.96 ± 136.12 | p<0.001 | n.s. (KO T55I) | p=0.003 (KO T55I) |
| KO T55I | 714.95 ± 61.26 | 110.26 ± 18.4 | p<0.001 | p<0.001 (WT) | p<0.001 (WT) |
| **Rotarod 20 RPM (Time-seconds)** | | | | | |
| WT | 499.81 ± 47.98 | 90.83 ± 7.48 | p<0.001 | n.s. (KO) | p=0.003 (KO) |
| KO | 461.86 ± 113.63 | 72.44 ± 14.7 | p<0.001 | n.s. (KO T55I) | p<0.001 (KO T55I) |
| KO T55I | 445.01 ± 59.87 | 42.65 ± 19.9 | p<0.001 | p=0.05 (WT) | p<0.001 (WT) |
| **Foot-slip test (number of miss-steps)** | | | | | |
| WT | 2.75 ± 0.96 | 7.69 ± 1.31 | p<0.001 | p<0.001 (KO) | p=0.008 (KO) |
| KO | 5 ± 1.2 | 10.16 ± 2.12 | p<0.001 | p=0.02 (KO T55I) | p<0.001 (KO T55I) |
| KO T55I | 6.3 ± 1.03 | 15 ± 2.54 | p<0.001 | p<0.001 (WT) | p<0.001 (WT) |

The average ± SD results for each genotype group are shown. *p* values obtained with the Student’s t-test and significant results *after Bonferroni correction* are shown for all comparisons (the group with which comparison was made is indicated in parentheses). n.s.: non-significant. RPM: rotations per minute.
